# Genome-wide association mapping of date palm fruit traits

**DOI:** 10.1038/s41467-019-12604-9

**Published:** 2019-10-15

**Authors:** Khaled M. Hazzouri, Muriel Gros-Balthazard, Jonathan M. Flowers, Dario Copetti, Alain Lemansour, Marc Lebrun, Khaled Masmoudi, Sylvie Ferrand, Michael I. Dhar, Zoë A. Fresquez, Ulises Rosas, Jianwei Zhang, Jayson Talag, Seunghee Lee, David Kudrna, Robyn F. Powell, Ilia J. Leitch, Robert R. Krueger, Rod A. Wing, Khaled M. A. Amiri, Michael D. Purugganan

**Affiliations:** 1 Center for Genomics and Systems Biology, New York University Abu Dhabi, Saadiyat Island, Abu Dhabi, United Arab Emirates; 2 Khalifa Center for Genetic Engineering and Biotechnology, UAE University, Al-Ain, Abu Dhabi, United Arab Emirates; 3 Center for Genomics and Systems Biology, New York University, New York, NY 10003 USA; 4 Arizona Genomics Institute, University of Arizona, Tucson, AZ 85705 USA; 5 Molecular Plant Breeding, Institute of Agricultural Sciences, ETH Zurich, 8092 Zurich, Switzerland; 6 Department of Evolutionary Biology and Environmental Studies, University of Zurich, 8057 Zurich, Switzerland; 7 Date Palm Research and Development Unit, UAE University, Al-Ain, Abu Dhabi, United Arab Emirates; 8 CIRAD, UMR Qualisud, F-34398 Montpellier, France; 9 Arid Land Department, College of Food and Agriculture, UAE University, Al-Ain, Abu Dhabi, United Arab Emirates; 10 Jardín Botánico, Instituto de Biología, Universidad Nacional Autonoma de Mexico, Ciudad Universitaria, Coyoacán, Mexico City, CP 04510 Mexico; 11 Royal Botanic Gardens, Kew, Richmond, Surrey, TW9 3AE UK; 12 United States Department of Agriculture-Agriculture Research Service, Riverside, CA 92507 USA; 13 Biological and Environmental Science and Engineering Division, King Abdullah University of Science and Technology, Thuwal, 23955-6900 Saudi Arabia; 14 Department of Biology, UAE University, Al-Ain, Abu Dhabi, United Arab Emirates

**Keywords:** Evolutionary genetics, Agricultural genetics, Genetic variation, Natural variation in plants

## Abstract

Date palms (*Phoenix dactylifera*) are an important fruit crop of arid regions of the Middle East and North Africa. Despite its importance, few genomic resources exist for date palms, hampering evolutionary genomic studies of this perennial species. Here we report an improved long-read genome assembly for *P. dactylifera* that is 772.3 Mb in length, with contig N50 of 897.2 Kb, and use this to perform genome-wide association studies (GWAS) of the sex determining region and 21 fruit traits. We find a fruit color GWAS at the R2R3-MYB transcription factor *VIRESCENS* gene and identify functional alleles that include a retrotransposon insertion and start codon mutation. We also find a GWAS peak for sugar composition spanning deletion polymorphisms in multiple linked invertase genes. MYB transcription factors and invertase are implicated in fruit color and sugar composition in other crops, demonstrating the importance of parallel evolution in the evolutionary diversification of domesticated species.

## Abstract (web summary)

Date palm is an important fruit crop in the Middle East and North Africa. Here, the authors report an improved genome assembly of this species and perform GWAS mapping of sex determining region and 21 fruit traits using high density SNP data generated from re-sequencing of the mapping population.

## Introduction

Domesticated crop species originated over the last 12,000 years through a co-evolutionary process as wild plant species are exposed to new selective environments associated with human cultivation and use^1^. There are ~1000 to ~2500 semi- and fully domesticated plant species^1^, and these species are largely responsible for providing food and fiber to human agricultural societies. There is great interest in dissecting the genetic and evolutionary mechanisms underlying domestication and diversification of crop species, both to illuminate general evolutionary principles as well as provide insights into the genetic basis of traits for crop improvement. Most genetic and evolutionary studies, however, have focused on annual crop species, primarily cereal crops. In contrast, much less is known about the evolutionary genetics of perennial crops such as fruit tree species.

The date palm (*Phoenix dactylifera* L.) is an iconic species and major crop of the Middle East and North Africa known for its sweet edible fruits. It is traditionally cultivated in oasis agrosystems in hot arid habitats that extend from Morocco to Egypt in North Africa, the Arabian Peninsula, Iraq, and Iran in the Middle East, and Pakistan and India in South Asia^2^. Date palms are a dioecious, obligate outcrossing, and highly heterozygous monocot species that are typically clonally propagated in agricultural contexts.

Date palms are one of the oldest domesticated perennial crops, with evidence of exploitation dating to ~7100 years before present (yBP) from Dalma Island, United Arab Emirates and As-Sabiyah, Kuwait^3^. Date palm cultivation is evident from the early Bronze Age (late 4th/early 3rd millennia BCE) in Mesopotamia and the Arabian peninsula, while it appears later in North Africa (end of 2nd millennium BCE)^3,4^. By the third millennium BCE, cuneiform texts in Sumerian, and later in Akkadian discuss date palm gardens in what is today Southern Iraq^3^.

The date palm holds an important place in the cultures of the Middle East and North Africa, appearing in major religious texts and iconography of the region. Date fruits were routine offerings in religious ceremonies in the 3rd millennium BCE and consumed in royal palaces together with figs, apples, and grapes. The Babylonian Code of Hammurabi (ca. 1754 BCE), one of the oldest legal texts in the world, prescribes regulations regarding date palm orchards^5^. In ancient Egypt, date palms were associated with the sun, and Rameses III (ca. 1186–1155 BCE) decreed that date gardens be planted in Heliopolis^6^. Theophrastus (ca. 350 BCE) discusses date palms in *Historia Plantarum*, and date palms were prominent in Phoenician and Carthaginian coinage ~2300 years ago.

After domestication, date palms diversified across the species range and today more than 3000 recognized varieties exhibit substantial variation in fruit-related traits such as color, size, moisture, and sugar content^7^. Early evidence of the diversification of fruit traits comes from archeological date stones which changed in size and shape consistent with selection for larger fruits^8,9^. Genomic data suggests that interspecific hybridization contributed to the diversification of cultivated date palms based on evidence of introgressive hybridization in North Africa between Middle Eastern cultivated date palms and the wild Cretan palm *P. theophrasti*^4^.

Date palms continue to be important to the economy and food security of the Middle East/North Africa. The importance of this crop has raised significant interest in the genetic basis of trait variation in this species both to aid future breeding efforts and understand the process of perennial crop domestication. However, long generation times (~4–6 years to first flowering), multiple years to generate offshoots for propagation, and 10–15 years to reach maximum yield all complicate multi-generation experiments^10^. This has hindered development of quantitative trait loci (QTL) mapping populations and efforts at breeding date palms for crop improvement. In contrast to other fruit crops, there are presently few populations suitable for QTL mapping in date palms, and breeding efforts are scarce or have been terminated^10^.

The difficulty of applying traditional genetic approaches has spurred the development of genomic resources to address problems in date palm cultivation and accelerate the discovery of important trait genes. Genome-wide association studies (GWAS) of tree crops provide an attractive alternative to QTL mapping approaches to identify loci controlling important traits^11,12^. In date palms, GWAS not only circumvent problems associated with long generation times, but high levels of nucleotide diversity and a relatively rapid decay of linkage disequilibrium (LD) [~20–30 kb]^4,13^ should enable high resolution mapping. GWAS mapping is facilitated by the availability of good quality genome assemblies, and in date palm two draft genome assemblies of ~690 Mb of the Khalas variety have been released^14,15^. However, published assemblies are highly fragmented with N50 of ~30 and 330 kb and ~57,000 and 82,000 sequence fragments, respectively^14,15^. Nevertheless, these genomes have enabled new discoveries^16^, including identification and characterization of the sex determination locus^14,17^, discovery of pathways active during fruit maturation^15^, and genome-wide studies of diversity^4,9,13^.

To facilitate GWAS in date palms, we generate an improved genome assembly from single molecule real time (SMRT) sequencing of a male date palm and conduct whole genome resequencing of a diverse set of male and female varieties located in two farms in the United Arab Emirates. The dense single nucleotide polymorphism (SNP) genotyping data from re-sequencing in conjunction with this assembly allow us to perform GWAS on key traits in this fruit crop that is of vital importance to the culture and economy of the Middle East and North Africa.

## Results

### Long-read sequence assembly of a date palm genome

**Fig. 1:**
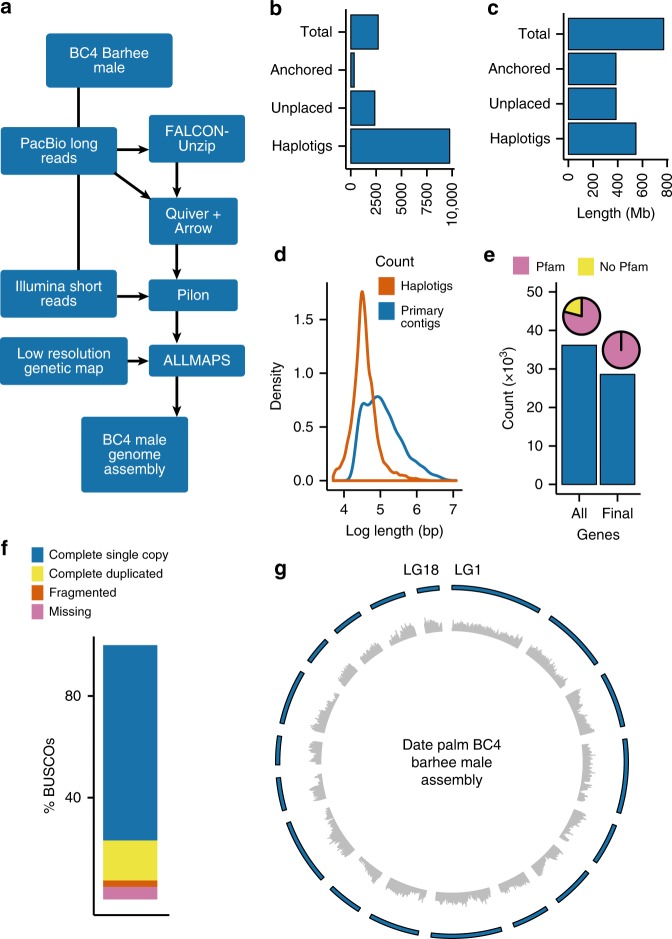
BC4 male date palm genome assembly. **a** Genome assembly work flow. **b** Total (anchored + unplaced) numbers of contigs. **c** Total (anchored + unplaced) contig lengths. **d** Length distribution of haplotigs and primary contigs generated by FALCON-Unzip. **e** Gene counts in the original (All) gene set and high confidence (Final) set. Pie diagrams indicate the percentage of genes with Pfam domains in each set. **f** Summary of BUSCO analysis of assembly completeness. **g** Circos diagram of 18 linkage groups showing gene density (inner) and linkage group ideograms (outer). Linkage groups are numbered in clockwise order beginning with LG1. Gene densities were calculated in sliding windows of 200 kb with step size of 50 kb. The source data underlying Fig. 1b–g are provided as a Source Data file

To improve on published date palm genome assemblies^14,15^, we use Pacific Biosciences (PacBio) sequencing to sequence a male date palm from a fourth-generation backcross (BC4) with a female of the Barhee cultivar (Fig. 1a, Table 1)^10,18^. We generated ~6.4 million long reads totaling 72 Gb of data (mean subread length = 11.2 kb, read length N50 = 18.5 kb) and supplement the PacBio sequences with ~110 million reads from 2 × 100 bp paired-end Illumina short insert libraries totaling 10.3 Gb of raw data. This results in a ~92.9× and ~13.8× fold-coverage of long and short reads, respectively (Supplementary Table 1).

**Table 1:** Statistics of the BC4 male date palm assembly and the previously published cultivar Khalas assemblies

Reference	Size (Mb)	Number of scaffolds or Falcon primary contigs	N50 (Kb)	Length of sequences anchored to LGs (Mb)
Al-Dous et al.^14^^*^	381	57,277	30.5	0
Al-Mssallem et al.^15^^**^	558	82,354	330.0	0
Present study	772	2,706	897.2	385.6

^*^GenBank reference number: GCA_000181215.2

^**^GenBank reference number: GCA_000413155.1

Using the FALCON-Unzip assembler^19^, we created a genome assembly with 2706 primary contigs and 9753 haplotigs (Fig. 1b, c, Supplementary Tables 1 and 2). The polished primary assembly spans 772.3 Mb and has high contiguity with contig N50 of 897.2 kb, while haplotigs consist of 547.4 Mb with N50 of 70.9 kb (Table 1, Fig. 1d, Supplementary Tables 1 and 2). From one-step flow cytometry, we estimated total genome size of *P. dactylifera* to be 870–899 Mb, indicating our draft assembly was 86–89% of the estimated size. We find our draft assembly is highly complete, with 98.05% of Illumina short reads, 94.2% of 7097 expressed sequence tags (ESTs) and >98% of RNA-Seq reads aligning to our BC4 male assembly (Supplementary Table 3). A BUSCO^20^ analysis of genome completeness indicates that >92.4% (1331 of 1440) of single copy ortholog groups in plants were recovered in the primary assembly, and only 5.1% were missing (Fig. 1e, f, Supplementary Table 4). Our assembly shows an increase in assembly size of ~18%, a ~2.7-fold increase in N50, and ~20-fold fewer assembly fragments compared to previous draft genome assemblies (Table 1)^14,15^.

We use a low-resolution genetic map^21^ to assign primary contigs to 18 linkage groups (LGs) in the date palm genome. Using ALLMAPS^22^, we anchored 49.9% (385.6 of 772.3 Mb) and orient 29.2% (225.8 Mb) of primary contig sequence to the 18 LGs (Supplementary Fig. 1). Linkage group 1 is the longest with 40.8 Mb of anchored sequence, and LG 18 the shortest with 9.8 Mb (Fig. 1g, Supplementary Tables 5 and 6). The ALLMAPS anchoring approach produces expected syntenic relationships between date palm LGs and oil palm pseudomolecules, including the observation that oil palm chromosome 2 is comprised of a probable fusion of date palm chromosomes 1 and 10^21^ (Supplementary Fig. 2).

We annotated the BC4 male assembly by generating RNA-Seq reads from leaf, fruit, root, pollen, and flower and predict genes with these data, publicly available ESTs, and UniProt proteins as input to MAKER2^23^ (Supplementary Table 7). We predict 36,162 non-transposable elements (TE) protein-coding genes and 51,395 gene models (including isoforms), but restricted further analysis to a high-confidence set (i.e., only genes containing a Pfam domain) consisting of 43,815 isoforms encoded by 28,595 genes (Supplementary Tables 1–8, Fig. 1e). The anchored assembly fraction was enriched in genes, with 66% of loci and 70% of isoforms located on the 18 LGs. Median gene size in the high-confidence set was ~4.2 kb, and median size of exons, CDS exons and introns were 159, 137, and 328 bps, respectively. Overall quality of the annotation was high, with 93.7% of high-confidence gene set models having an annotation edit distance (AED) score^23^ of 0.5 or lower (79.9% for the whole set, Supplementary Table 8). We annotated the repeat fraction of the genome and found more than half of the BC4 male assembly consists of repeats and TEs, with long-terminal repeat retrotransposons (LTR-RTs) occupying most of the TE space (Supplementary Table 9), as reported for date palm and other palm species^15,24^.

### GWAS mapping of the sex determination locus

Increased assembly contiguity provides a unique resource for genome investigation and agricultural genomics research, including the ability to conduct GWAS mapping in date palms. To test the utility of our improved assembly, we used GWAS to map the sex determining region characterized previously^14,17^.

**Fig. 2:**
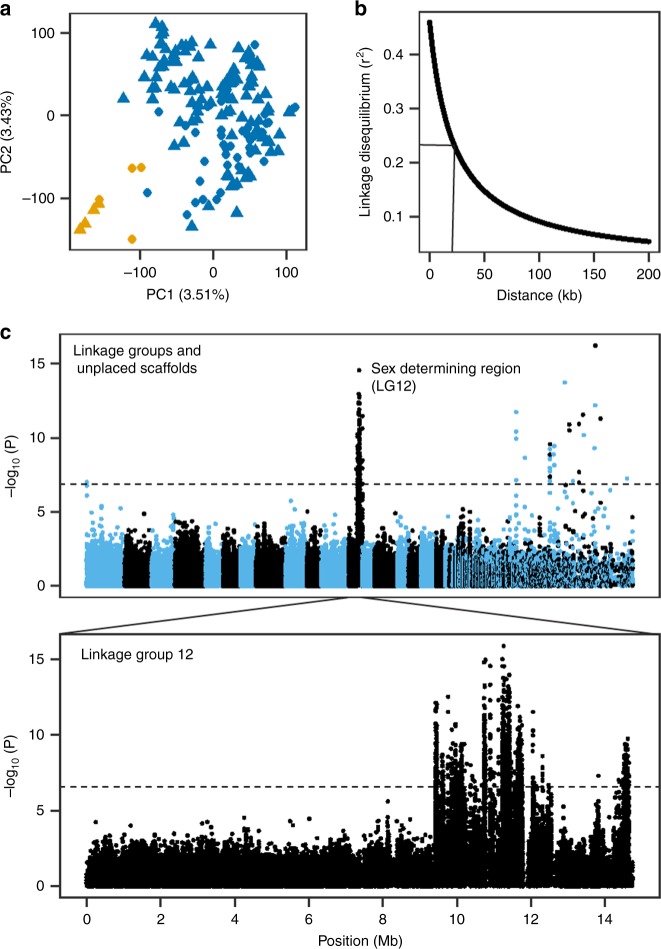
GWAS analysis of the sex determination region of date palm. **a** PCA of genetic structure in the GWAS panel. Points are colored based on whether the ancestry of the variety traces to Middle Eastern (blue) or North African (orange) date populations. The shapes of the points indicate the location of the tree in Al-Shuwaib (triangle) or Ras Al-Khaimah (dot), **b** decay of linkage disequilibrium with physical distance in the GWAS mapping population. The half decay distance is 22.9 kb, **c** GWAS of sex determination in date palm (*n* = 157 date palms). Top: Manhattan plot using the randomly downsampled SNP set (392,948 SNPs) for all linkage groups and unplaced scaffolds, below: Manhattan plot using the full SNP set on LG 12. Corresponding quantile-quantile plot can be found in Supplementary Fig. 4. Source data are provided as a Source Data file

We characterized this trait in a GWAS panel comprised of date palms grown in two farms located in Al-Shuwaib, Abu Dhabi and Hamriyah, Ras Al-Khaimah, United Arab Emirates (Supplementary Data 1). Together, they comprise a diverse mapping population of 145 female varieties and 12 male individuals without obvious population structure related to the two farms (Supplementary Fig. 3, Supplementary Data 1). We used paired-end (2 × 100 bp) Illumina sequencing to re-sequence genomes of the 157 date palms to an average depth of ~11.3× (Supplementary Data 1). Following application of SNP quality control filters, we identified 7,149,205 SNPs or approximately 9.24 SNPs/kb, in our GWAS panel. We performed compressed mixed linear model (CMLM) GWAS using both the first five principal components of population structure and kinship information as covariates^25,26^ (Fig. 2a; Supplementary Data 2 and 3). Since LD decays relatively rapidly in our GWAS panel (half decay distance = 22.9 kb, Fig. 2b), we chose not to remove LD-correlated SNPs. Instead, we randomly sample 392,948 SNPs to yield ~1 SNP every 2 kb for mapping. We applied a conservative Bonferroni threshold to identify significant SNPs, and ensure that all GWAS models with significant associations had a genomic inflation factor λ of 1 ± 0.15 in quantile–quantile plots.

Using these criteria, we are able to map the date palm sex determination region to the distal end of LG 12 consistent with previous reports^14,17,21^ (Fig. 2c, Supplementary Fig. 4). The GWAS peak consists of 112 SNPs that span a region of ~6 Mb of placed primary contig sequence on LG 12, and 43 additional significant SNPs located on unplaced contigs (Supplementary Datas 4 and 5). The large section of LG 12 associated with sex is consistent with reduced recombination within the sex determining region (and thus high LD). It should be noted that a high-resolution view of this region reveals two peaks (Fig. 2c), but it is unclear if these are independent peaks or represent a statistical or assembly artifact.

### GWAS mapping of date palm fruit traits

Given our success in mapping the sex determination region, we conducted GWAS on 21 fruit traits, many of which distinguish commercial cultivars (e.g., fruit color and sugar composition) and are important in the evolutionary diversification of date palms (Supplementary Data 1). We phenotyped two fruit size (length and weight), eight fruit color (seven color parameters and anthocyanin level), fruit moisture, four fruit sugar content (sucrose, glucose, fructose, and percentage of sucrose to total sugar) and six acid level (fumaric, citric, malic, succinic, oxalic, and tartaric acids) traits (Supplementary Fig. 5). Our analysis reveals no significant associations for most traits, including fruit size and acid content, suggesting these traits may require significantly larger mapping populations to successfully identify genes underlying these traits (Supplementary Fig. 6). However, mapping of fruit color and sugar composition reveal significant GWAS hits.

### Genetics of fruit color in date palms

**Fig. 3:**
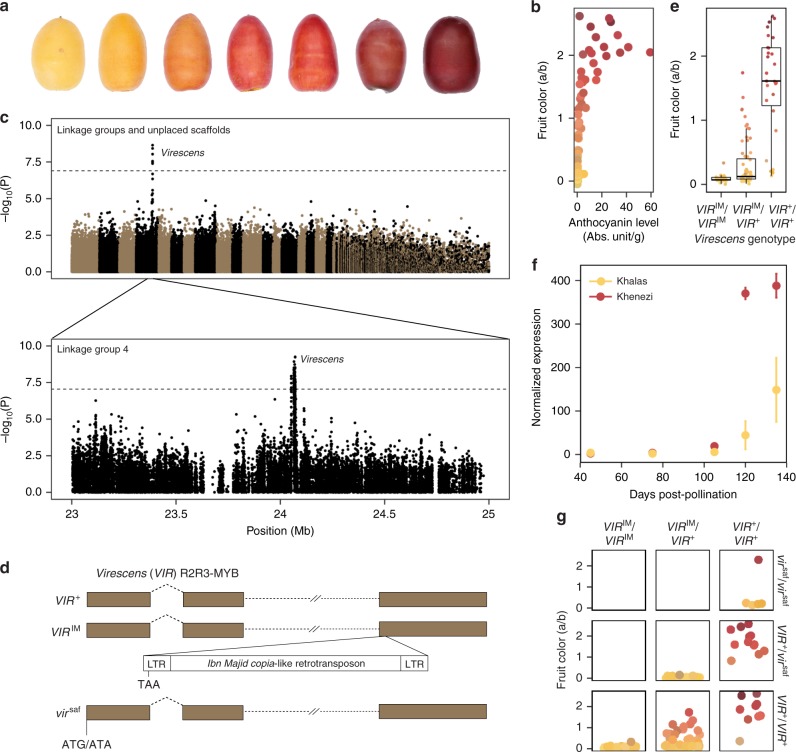
The genetics of fruit color variation in date palms. **a** Color variation in khalal stage fruits among date palm varieties. **b** Fruit color measured by a color index (**a**, **b**) as a function of anthocyanin level with points colored according to the RGB score of the fruit color. Each point represents the average of eight fruits per cultivar. **c** GWAS of fruit color in date palms (*n* = 157). Top: Manhattan plot using the randomly down-sampled SNP set (392,948 SNPs) for all linkage groups and unplaced scaffolds. Below: Manhattan plot using the full SNP set on LG 4. The dotted lines in each panel represent the Bonferroni significance threshold. **d** Gene models of *VIR* alleles. The *VIR*^IM^ allele is shown with the *Ibn Majid* retrotransposon insertion which introduces a premature stop in exon 3 and *vir*^*saf*^ allele is shown with the start codon polymorphism caused by a G to A change. **e** Boxplot distributions of fruit color phenotypes by *Ibn Majid* genotype (*n* = 145 date palms). Center line is the median, bounds of box represent the frst and third quartiles, the upper and lower whiskers extend from the hinge to the largest or smallest value, respectively, no further than 1.5 * IQR from the hinge (where IQR is the inter-quartile range, or distance between the first and third quartiles). **f** RNA-Seq analysis of *VIR* expression across fruit development in two varieties with red and yellow fruits. Each point is the mean of normalized expression counts across three or more replicates. Error bars represent s.e.m. **g** Variation in fruit colors by two-locus genotype. Loci are defined by the *Ibn Majid* and start codon mutations in *VIR*. Points in **e**–**g** are colored as in (**b**). Points in **g** are jittered randomly on the *x*-axis. The source data underlying Fig. 3b, c, e–g are provided as a Source Data file

Date palm fruits vary in color from deep red to pale yellow in the fresh (khalal) and ripe (rutab) stages (Fig. 3a). To measure fruit color, we use the RGB color space model [measuring the strength of red (R), green (G), and blue (B)] from scanned images of fruits^27^, and the Commission Internationale de L’Eclairage (CIE) model, which measures lightness (L), red/green (a), and yellow/blue (b) strengths from photographs. Principal component (PC) analysis on all color-related measurements indicated separation on PC1 for red/yellow colors and PC2 for yellow intensity (Supplementary Fig. 7).

To map fruit color, we focus on the ratio of a/b, where higher values represent greater red coloration and lower values indicate yellow (Fig. 3b). We find significant associations (*F*-test, *P* < 1.3 × 10^−7^) in a region on LG 4, and no other significant associations (Fig. 3c, Supplementary Fig. 4, Supplementary Data 5). A separate analysis using all SNPs on LG 4 indicate that the GWAS peak spans a region of ~20 kb from 24.052 to 24.073 Mb (Fig. 3c). This region contains a TOC75-3 chloroplastic-like gene, a gene of unknown function, and the orthologue of the oil palm *VIRESCENS* (*VIR*) gene that encodes an R2R3-MYB transcription factor that underlies fruit color in oil palms^28^ (Supplementary Table 10). Mapping the R and G color levels from the RGB model, and a, b, and L parameters from the CIE system also yielded significant associations in the same region (Supplementary Data 5, Supplementary Fig. 8). Our GWAS approach therefore confirms our previous candidate gene analysis^13^ that suggested that the ortholog to oil palm *VIR* controls the red/yellow fruit color polymorphism in date palms.

We previously characterized the date palm *VIR* gene and reported a retrotransposon insertion polymorphism that interrupts exon 3 and introduces a premature stop codon at position 169 of the translated protein^13^. Examination of the BC4 male genome indicates our genome assembly also contains this retrotransposon insertion allele (Fig. 3d, Supplementary Table 11). However, in contrast to an earlier draft genome^15^, the long-read assembly contains a complete copy of the *copia*-like retrotransposon that is ~11.7 kb in length, has 469-bp long terminal repeats, has a 5-bp target site duplication at its insertion site, and flanked by sequences homologous to exon 3 of oil palm *VIR* on each side of the insertion (Fig. 3d, Supplementary Table 11). This retrotransposon, which we name *Ibn Majid*, after the 15th-century Arab navigator^29^, is found in low copy numbers in the date palm genome, and shows only weak similarity to other plant *copia*-like retrotransposons.

We genotyped the retrotransposon insertion polymorphism in our GWAS panel and evaluate fruit color and anthocyanin content in each genotypic class. We observe that homozygotes for the *Ibn Majid* retrotransposon allele, hereafter *VIR*^IM^ (Fig. 3d), invariably have yellow fruits (Fig. 3e). In contrast, homozygotes for the wild type allele (*VIR*^+^) have predominantly red fruit, while heterozygous varieties have yellow fruits or fruits with intermediate colors including shades of orange. The fruit color patterns are consistent with what we observe in anthocyanin content: *VIR*^IM^/*VIR*^IM^ homozygotes produce undetectable amounts of anthocyanin, *VIR*^+^/*VIR*^+^ have predominantly high concentrations, and *VIR*^+^/*VIR*^IM^ produce undetectable or trace amounts of anthocyanin (Supplementary Fig. 9). This suggests that *Ibn Majid* disrupts an activator of anthocyanin biosynthesis similar to oil palm truncation alleles at the *VIR* locus^28^. While anthocyanin content in these genotypic classes are consistent with functional variation at *VIR*, GWAS on anthocyanin content does not lead to significant hits, possibly due to greater variability of anthocyanin level measurements that result in reduced power to detect GWAS peaks.

Analysis of fruit color phenotypes of putative *VIR*^+^/*VIR*^+^ homozygotes revealed four varieties that produce yellow fruits despite the absence of the retrotransposon insertion (Fig. 3e). We observe that these four varieties, including the common Saudi Arabian variety Safri (=Sufri), are homozygous for a SNP that changes the predicted translation initiation codon of *VIR* from ATG to ATA (Fig. 3d) at position 24,051,180 of LG 4 in the BC4 male assembly (Supplementary Table 11). No other in-frame methionine codons are found immediately upstream or downstream of this position in *VIR*^+^ suggesting no alternate translation initiation sites. This suggests that the loss of a translation initiation codon likely results in a loss-of-function allele, hereafter *vir*^saf^, at the *VIR* locus.

We collected RNA-Seq data to determine how wild type and *VIR*^IM^ alleles are expressed during fruit development. RNA-Seq analysis reveals that date palm *VIR* is expressed late in developing fruits, peaking at 120–135 days post pollination (dpp) in cultivars with both red (Khenezi variety) and yellow (Khalas) fruits (Fig. 3f). Our analysis confirms that *VIR*^IM^ is expressed and that Khalas has reduced expression compared with Khenezi at 105, 120, and 135 days post pollination (Supplementary Table 12).

The red color of *VIR*^+^/*vir*^saf^ fruits implies that *VIR*^+^ is haplosufficient, and *vir*^saf^ is recessive consistent with it being a loss-of-function allele (Fig. 3g). In contrast, *VIR*^+^/*VIR*^IM^ heterozygotes have yellow or orange fruits in which *VIR*^IM^ acts as a dominant, or possibly semidominant, negative inhibitor that interferes with the expression of the wild-type *VIR*^+^ allele. This genetic behavior is similar to mutants of other R2R3-MYB transcription factors such as maize *C1*^30^. Thus red fruits are produced by *VIR*^+^/*VIR*^+^ and *VIR*^+^/*vir*^saf^, yellow fruits by *vir*^saf^/*vir*^saf^, *VIR*^IM^/*VIR*^IM^, *VIR*^IM^/*vir*^saf^, and some *VIR*^+^/*VIR*^IM^ genotypes (Fig. 3g, Supplementary Fig. 10) and intermediate colors by *VIR*^+^/*VIR*^IM^ genotypes, with the action of the *VIR*^+^/*VIR*^IM^ genotypes due to dominant negative inhibition of anthocyanin production.

### Genetics of fruit sugar composition

**Fig. 4:**
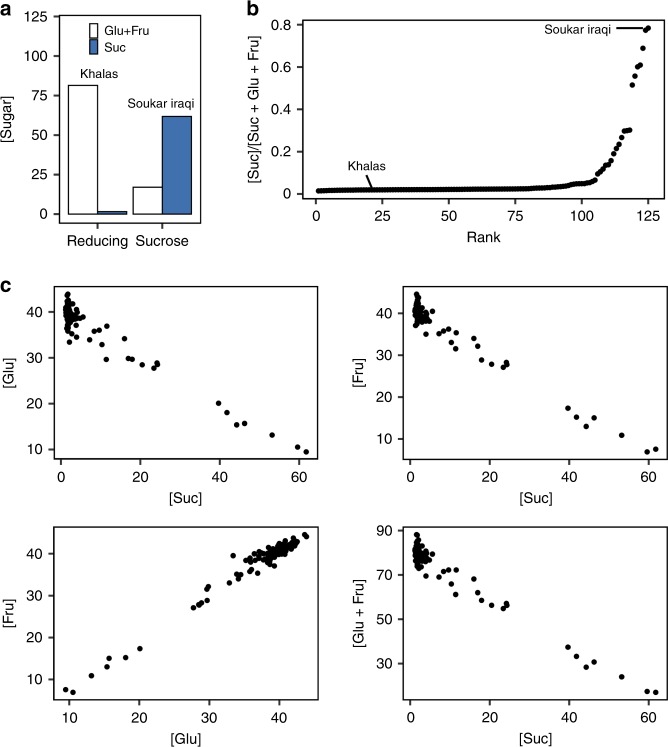
Date palm fruit sugar composition phenotypes in the GWAS panel. **a** Sugar composition (g/100 g dry matter) of reducing-type and sucrose-type varieties Khalas and Soukar Iraqi at the tamar stage (Suc = sucrose, Fru = fructose, Glu = glucose). **b** Distribution of the percentage sucrose to total sugar in the mapping population. **c** Correlations of sugar concentrations across varieties. Source data are provided as a Source Data file

Sugar composition is a characteristic of date fruits that varies among cultivars and contributes to the distinctive flavor profile of individual varieties. During kimri and khalal stages of fruit development, sucrose and starch accumulate in the mesocarp of developing date fruits. As the fruit matures, sucrose is hydrolyzed into the reducing sugars fructose and glucose to varying degrees, dependent on the variety. Relative concentrations of sucrose, fructose and glucose are used to classify date palm varieties as either sucrose type with high concentrations of sucrose at the tamar stage (the stage when date fruits have low-moisture content and are typically consumed) or reducing-sugar type defined by high concentrations of glucose and fructose^31^ (Fig. 4a, b).

We quantified sucrose, glucose, and fructose at tamar stage fruits, and observed a wide range of sugar composition across varieties. Most samples, including the Khalas variety, are of the reducing-sugar type with high concentrations of glucose and fructose and a low percentage of sucrose to total sugar ([sucrose]/[glucose + fructose + sucrose]) (Fig. 4a, b). Sucrose-type varieties with high sucrose concentrations are less common and include the highly prized Sukkari (=Soukar/Soukari which in English is translated as sweet) varieties (Fig. 4a, b). We find concentrations of these three sugars are strongly correlated, with sucrose–glucose, sucrose–fructose, and sucrose–(glucose + fructose) being strongly negatively correlated (Fig. 4c, Supplementary Table 13). Thus, date palm genotypes with increased amounts of sucrose also have decreased levels of both glucose and fructose.

**Fig. 5:**
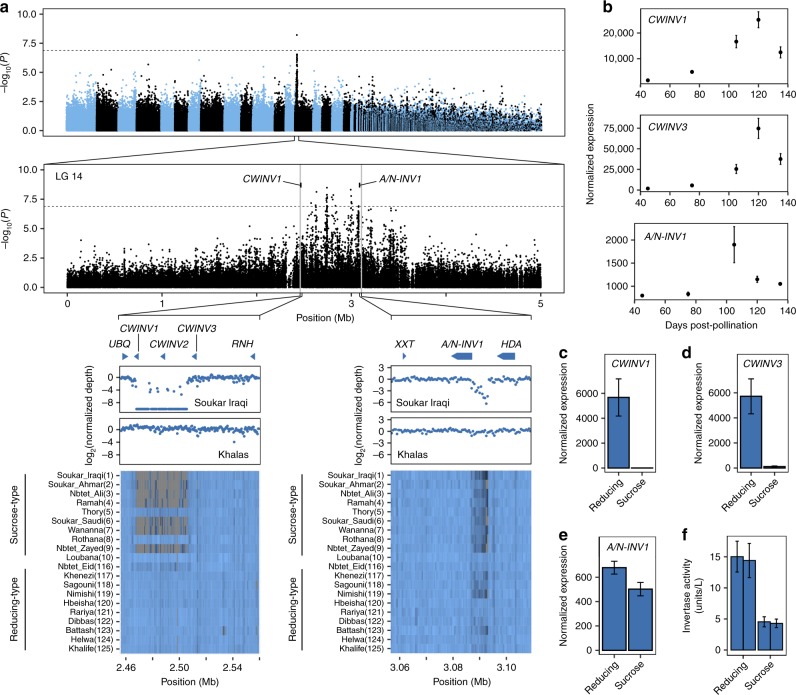
GWAS mapping of sugar composition in date palm fruit and functional characterization of candidate genes. **a** Top: Manhattan plot GWAS results for tamar stage fruit sugar composition measured as [sucrose]/[sucrose + glucose + fructose]. The plot is based on analysis conducted with the randomly downsampled SNP call set. Middle: Manhattan plot of the region on LG 14 containing significant associations based on analysis of all SNPs on this linkage group. Dotted lines represent the Bonferroni significance threshold. Gray vertical lines represent boundaries of the regions highlighted below. Bottom: Close-up of two highlighted regions with invertase genes in the vicinity of the GWAS peak. From top to bottom, gene models, normalized coverage depth in 500 bp windows for a sucrose-type variety (Soukar Iraqi), a reducing sugar-type variety (Khalas), and a heatmap of normalized coverage depth (low coverage is dark blue to high coverage light blue; zero coverage = gray) of the ten most extreme sugar composition phenotypes at each end of the sucrose-type and reducing-type distributions (see Supplementary Figs. 12 and 13 for all samples). Numbers in parenthesis next to variety names are the sucrose to total sugar rank among the 125 varieties with sugar measurements, where (1) is highest sucrose. **b** Expression of three candidate invertase genes in developing fruit at different stages post pollination (mean ± s.e.m.) in the Khenezi variety. **c**–**e** Differential gene expression of khalal stage fruit between four sucrose-type and four reducing-type varieties at three candidate invertase genes (mean ± s.e.m.). **f** Invertase enzyme activity in khalal stage fruit in two reducing-type and two sucrose-type varieties (mean ± s.d.). Source data are provided as a Source Data file

We mapped levels of sucrose, glucose, and fructose and percent sucrose to total sugar [sucrose]/[sucrose + glucose + fructose], and found all four traits share a common GWAS peak on LG 14 (*F*-test, *P* < 1.3 × 10^−7^) [Fig. 5a, Supplementary Fig. 11, Supplementary Data 5]. Separate analysis restricted to SNPs on LG 14 indicates that SNPs significantly associated with these traits span a broad region of ~1.1 Mb from approximately positions 2.4 to 3.6 Mb (Fig. 5a). Of the 162 annotated protein-coding genes in this region in the BC4 male assembly (Supplementary Data 6), three encode invertase (β-fructofuranosidase) enzymes, which function to hydrolyze sucrose into fructose and glucose and function in sugar accumulation in fruits from a range of species^32^. The positional invertase candidates include an alkaline/neutral invertase (*A/N-INV1*; gene ID chr14G0028200) located near the center of the GWAS peak at 3.087 Mb of the LG 14 assembly (Fig. 5a, Supplementary Data 6), and two adjacently-linked cell wall invertases (*CWINV1* and *CWINV3*, gene ID chr14G0022900 and chr13G0023100, respectively) and located at the 5′ end of the GWAS region at approximately 2.469 and 2.512 Mb (Fig. 5a).

The CWINV1 and CWINV3 proteins show 98.58% amino acid identity, suggesting these are tandem duplicates of each other. We check for additional un-annotated copies of invertase in this region by aligning predicted transcripts for each of the three genes to this region, and recovered a minus strand sequence (which we refer to as *CWINV2*), with close homology to *CWINV1* and *CWINV3*, and located between these two genes at positions 2,489,373 to 2,485,592 on LG14 (Fig. 5a). However, this coding region has multiple frame-shift mutations in our PacBio assembly and may be a pseudogene in the BC4 male individual. Examination of the whole genome re-sequencing data suggests that many of these frameshift mutations in *CWINV2* are not present in other date palm samples, and it is unclear if this gene is functional in at least some varieties. Nevertheless, we do not consider *CWINV2* further in our analyses, although we note that the functional status of this locus does not materially affect our conclusions (see below).

We scanned the fruit sugar composition GWAS region for candidate major effect SNPs, small coding insertion/deletion (indel) polymorphisms and copy number variants that might explain sugar composition variation. A sliding window analysis of normalized coverage depth in this region found a polymorphic deletion of ~40 kb at approximately 2.467 to 2.507 Mb that includes the 5′ half of *CWINV1* (including the first two coding exons and its promoter region), a region ~5 kb downstream of *CWINV3*, and the putative *CWINV2* pseudogene (Fig. 5a). The extent of this deletion renders *CWINV1* nonfunctional. A second polymorphic deletion of ~5 kb is located closer to the center of the GWAS peak between approximately 3.088 and 3.093 Mb in noncoding sequence between the 5′ end of *A/N-INV1* and an adjacent histone deacetylase (HDA) gene (Fig. 5a).

Examination of coverage depth data in the *CWINV1/3* and *A/N-INV1* deletion regions suggests that sucrose-type varieties, such as Soukar Iraqi, are more frequently homozygous for these deletions than reducing-type varieties such as Khalas (Fig. 5a, Supplementary Figs. 12 and 13). We find that homozygotes for the deletion at *CWINV1/3* have higher sucrose concentration than alternate genotypes (one-sided Wilcoxon-rank sum test, *W* = 1264, *P* < 9.3 × 10^−7^) as do homozygotes for the deletion in the promoter region of *A/N-INV1* (*W* = 855*, P* < 3.8 × 10^−5^).

RNA-Seq analysis of invertase genes across date palm fruit development shows that all three invertases are expressed in the fruit and that *A/N-INV1* peaks in expression at ~105 days post pollination, whereas *CWINV1* and *CWINV3* peak at ~120 days (Fig. 5b). Tests of RNA levels between khalal stage fruit (ca. 120 days after pollination) of sucrose- and reducing-type varieties indicate that both *CWINV1* (Wald test, *P* < 1.25 × 10^−16^) and *CWINV3* (*P* < 3.06 × 10^−19^) are differentially expressed (Fig. 5c, d, Supplementary Table 14). Log_2_ fold-changes between sucrose- and reducing-type varieties are 2.69 and 4.23 for *CWINV1* and *CWINV3*, with higher expression in reducing-types suggesting a large difference in gene expression in developing fruits (Supplementary Table 14). Reduced expression in both *CWINV1* and *CWINV3* is consistent with the 40-kb deletion removing much of *CWINV1*, including its promoter region and possibly affecting a 3′ regulatory region of *CWINV3*, although we cannot exclude the possibility that there is additional functional variation that alters the regulation of this gene. In contrast, *A/N-INV1* shows a much weaker difference in expression between fruit types (Wald test, *P* < 0.08, log2 fold-change = 0.41, Fig. 5e, Supplementary Table 14). Finally, a test of differences in invertase enzyme activity between the two sugar types find that sucrose-type varieties have significantly lower activity (two-sided *t* test, *t* = 15.001, d*f* = 2, *P* < 0.0044) (Fig. 5f, Supplementary Table 15) at this stage as reported previously^33^.

The GWAS association in a region with multiple invertase genes, prevalence of invertase gene deletions in sucrose-type varieties, and negative correlation between sucrose and hexose sugars across varieties (Fig. 4c, Fig. 5, Supplementary Data 6), are consistent with fruit sugar composition being controlled by functional variation at one or more invertase genes. These observations support a model where gene-deletion polymorphisms and/or altered gene regulatory regions reduce expression of one or more fruit-expressed invertases, which limits sucrose hydrolysis at advanced stages of development of sucrose-type fruits. It remains unclear whether only the cell wall invertases (*CWINV1*, *CWINV3*), the alkaline/neutral invertase (*A/N-INV1*), or both control sugar composition in date palm fruit. Moreover, we are uncertain if the *CWINV2* pseudogene also impacts this trait (e.g., if it is functional in some varieties), but we note that this locus is found in the middle of the 40-kb deletion that spans the putative regulatory regions of *CWINV1* and *3* and deletes coding exons of *CWINV1*. Thus, its functional status does not affect our hypothesis that this deletion is the major candidate causal polymorphism. We also note that the cell wall invertases and the *A/N-INV1* genes are ~600 kb apart, which may explain the wide (~1 Mb) GWAS peak for fruit sugar composition despite the outcrossing nature of date palms, and suggests that both cell wall and alkaline/neutral invertase types contribute to variation in this trait.

Finally, while these deletions are clearly correlated with both sucrose/reducing sugar and invertase transcript levels, the correlations are not perfect; indeed, some sucrose-type varieties do not contain the *CWINV1/3* deletion and some of the reducing sugar varieties contain the *A/N-INV1-*associated deletion. This suggests that either there are other, as yet unidentified, causal polymorphisms (such as seen in the independent start codon mutation in the *vir*^saf^ allele and fruit color), or other loci may be contributing to this quantitative trait. Thus, although our data provides strong support for one or more invertases on LG 14 being responsible for sugar composition in date palm fruits, and that large deletions at these loci are associated with reduced invertase activity, further molecular genetic study will be needed to fully dissect the functional underpinnings of this trait.

## Discussion

Date palms are a key crop of the Middle East and North Africa, and are a mainstay of arid land agriculture. Despite its economic and cultural importance, genetic studies of date palm are limited by relatively few genomic resources and challenges posed by working with tree species with long generation times. To help remedy this situation, our long-read sequencing approach has produced an improved genome assembly for date palms that is 18% larger and is more contiguous than two current draft reference genome assemblies based on short-read sequencing^14,15^. This long-read genome sequence assembly, coupled with access to two mature date palm orchards in the United Arab Emirates with 157 male and female individuals, has allowed us to conduct genome-wide association mapping in this species.

We successfully mapped the previously identified sex determination locus^14,17^, as well as genes for both fruit color and sugar level polymorphisms. However, our relatively small GWAS panel of 145 female samples allowed us to map only 11 of 21 fruit traits, many of which were correlated with each other. If phenotypic variation in the unmapped traits are genetically determined, future mapping efforts will require significantly larger mapping populations, which may be difficult in the near term given we currently do not know any date palm orchard in the world with larger numbers of different date palm cultivars than the one we used in this study.

Our results support the idea that key domestication and diversification traits are driven by evolutionary convergence in homologous genes across unrelated taxa. We find that fruit color polymorphism in date palms is caused by mutations in a gene encoding an R2R3-MYB transcription factor. Homologous genes in grape^34^, apple^35^, cacao^36^, citrus^37^, and other perennial crops across different plant families are also responsible for fruit color variation in those species. We have also mapped fruit sugar content to a genomic region that includes invertases that segregate deletion polymorphisms in the date palm population. The cell wall invertase genes show large expression differences between varieties with different fruit sugar compositions, suggesting these may be play a causal role in this trait. Natural variation of invertase is also implicated in sugar composition of tomato^38^ and grapes^39^ and suggests an important role for parallel evolution during diversification of domesticated fruit crops. Interestingly, for both fruit color and sugar composition, structural mutations (a retrotransposon insertion in *VIR* and kilobase-sized deletions in the invertase genes) appear to be primarily responsible for phenotypic changes.

Our results support previous studies which show that phenotypic convergence/parallelism is a hallmark of crop domestication, a result of similar selection pressures acting on different crop species across distinct human cultures^40^. Indeed, the parallel evolution of traits among domesticated species was noted by N. I. Vavilov, who in 1922 proposed the genetic law of homologous series of variation among related crop species^41^. As we continue to uncover the genetic basis for crop evolution, we may compare and contrast genetic architectures across the entire spectrum of domesticated taxa, and discover similarities in the evolutionary trajectories even between annual and perennial crops despite their marked differences in life history.

## Methods

### Genome sequencing of the BC4 male

We sampled a backcross male date palm located at the United States Department of Agriculture (USDA)/University of California, Riverside farm in Thermal, California (USDA accession No. PI 555415, Source RIV 7545 PL). This male was produced by four generations of backcrossing with a Barhee female as the recurrent parent as part of a breeding program at the USDA, USA that was discontinued in the 1970s^10,18^. Leaflets were cleaned and snap frozen on liquid nitrogen prior to transport to the Arizona Genomics Institute (University of Arizona, Tucson, AZ) for extraction of high molecular weight DNA and sequencing.

The genome of the BC4 male was sequenced using a PacBio RSII sequencing platform. High molecular weight DNA for sequencing was extracted from young leaves adopting the protocol of Doyle and Doyle^42^ with minor modifications. PacBio library preparation followed the 20 kb protocol [http://www.pacb.com/wp-content/uploads/2015/09/User-Bulletin-Guidelines-for-Preparing-20-kb-SMRTbell-Templates.pdf] and three libraries (gel-selected at 20, 25, and 30 kb) were built. Eighty-five SMRT cells were sequenced on a RSII sequencer with movie collection time of 6 h. About 6.4 million reads were generated, totaling 72 Gb of data (mean subread length 11.2 kb, N50 18.5 kb). Additional sequencing of a short insert library (2 × 100 bp paired-end) was conducted with an Illumina HiSeq 2500 sequencer.

### Genome assembly

We did a k-mer-based estimation of the genome size from raw short read sequences of the BC4 male genome for assembly purposes (KmerFreq_AR in SOAPdenovo2^43^) with default settings and k-mer length set to 17. Note that an experimental genome size estimate for *P. dactylifera* was also done using flow cytometry (see below). PacBio reads were then assembled with FALCON-Unzip^19^ (v. falcon-2017.06.28–18.01-py2.7-ucs2) with a seed coverage of 55× and the k-mer-based genome size estimate of 774 Mb as input. The Unzip module was run with default settings.

The resulting assembly was polished by aligning raw PacBio reads with Quiver and Arrow (part of the SMRT Analysis suite v. 2.3.0) followed by running Pilon^44^ v. 1.18 with Illumina short read sequences from the BC4 male. Inputs to Pilon were produced by trimming the short reads with Trimmomatic^45^ (v. 0.32) to remove 3′ bases below base quality of Q30 and reads shorter than 30 nucleotides. Reads were then aligned to the output of Arrow with Bowtie2^46^ (v. 2.2.6).

The polished primary contigs were anchored to LGs of the existing genetic map^21^ with ALLMAPS^22^ to produce an anchored haploid assembly. Scaffold sequences for the genetic map were obtained from http://qatar-weill.cornell.edu/research/datepalmGenome/edition3/PdactyKAsm30_r20101206.fasta.gz. Upon alignment to the genetic map and after manual inspection of the realignment of the raw reads to the assembly, we found only one instance of mis-assembly: one contig had to be split since two contig ends were merged head-to-head.

### Genome annotation

We generated RNA-Seq libraries from multiple khalal stage fruit (see below), a mixture of male and female flower buds (referred to as “flower” below), and pollen, and conducted 2 × 100 bp paired-end sequencing on an Illumina HiSeq 2500 instrument (Supplementary Table 7). Additional date palm RNA-Seq data from leaf and root were downloaded from the Sequence Read Archive [https://www.ncbi.nlm.nih.gov/sra] (Supplementary Table 7). RNA-Seq reads were trimmed with Trimmomatic^45^, aligned to the haploid assembly with STAR^47^ (v.2.4.0.1), and gene models predicted by StringTie^48^ (v. 1.3.2) to be used as training for Augustus^49^ (v. 2.3).

Gene annotation was performed using the MAKER2 pipeline^50^ (v. 2.31). Homology-based evidence, included 7097 ESTs (downloaded from NCBI EST database on February 9, 2017), protein sequences from Uniprot^51^, a date palm proteome [http://qatar-weill.cornell.edu/research/research-highlights/date-palm-research-program/date-palm-genome-data], an oil palm proteome^52^, and the RNA-Seq derived models from above. Ab initio prediction was performed with Augustus (v. 3.0) trained as described in Bowman et al.^53^ with gene models produced with StringTie^48^ (v. 1.3.2), from the RNA-Seq alignments.

The raw MAKER2 annotation was parsed, removing models containing TE domains and lacking evidence of transcription or the presence of a Pfam domain as described in Bowman et al.^53^. With about 1× of non-organellar single-end WGS Illumina reads, a de novo (non assembly-based) repeat library was produced with RepeatExplorer^54^, and parsed as in Copetti et al.^55^. Repeat annotation of the assembly was performed with RepeatMasker (v. 4.0.6; [http://www.repeatmasker.org/] in nucleotide space) and Blaster^56^ (part of the REPET v 2.5 package, in protein space) and later reconciled in a single annotation file. Noncoding RNAs were predicted with Infernal^57^ (v. 1.1.2) with the Rfam library^58^ (v. 12.2). Hits above the *e*-value threshold of 1 × 10^−5^ were filtered out, as well as results with score lower than the family specific gathering threshold. When loci on both strands were predicted, only the hit with the highest score was kept. Transfer RNAs were also predicted using tRNAscan-SE^59^ (v. 2.0) with default parameters.

### Genome quality assessment

Visualizations of the genome assembly were produced with assembly-stats software (Supplementary Fig. 1, [https://assembly-stats.readme.io/docs]). Assembly completeness was evaluated by characterizing the gene space with BUSCO^20^ using 1440 plant ortholog groups (v. 3) and by aligning ESTs to the diploid assembly with Blat^60^ (v. 350).

### Date palm genome size estimation

The genome size was estimated using the one-step flow cytometry procedure described in Doležel et al.^61^ with slight modifications. Briefly, approximately 1 cm^2^ of leaf material from two *P. dactylifera* samples at the Royal Botanic Gardens, Kew, UK collection was incubated for 30 s on ice in 1 ml of “general purpose buffer” (GPB)^62^ supplemented with 3% PVP-40 to soften the leaf. Then a similar amount of leaf material of the calibration standard *Petroselinum crispum* (Mill.) Fuss (1C value = 2201 Mb)^63^ was added and the combined material was chopped rapidly (but not too vigorously) using a new razor blade. A further 1 ml of the GPB buffer was added and then the homogenate was filtered through a 30 µm nylon mesh (Celltrics 30 µM mesh, Sysmex, Goritz, Germany) into a tube, 100 μl propidium iodide (1 mg/mL) was added, and the sample was incubated on ice for 10 min. The relative fluorescence of 5000 particles was recorded using a Partec Cyflow SL3 flow cytometer (Partec GmbH, Münster, Germany) fitted with a 100 mW green solid-state laser (532 nm, Cobolt Samba, Solna, Sweden). Three replicates of each leaf were processed, and the output histograms were analyzed using the FlowMax software v.2.4 (Partec GmbH). The 1C value of *P. dactylifera* (Mbp) was calculated as: (Mean peak position of *P. dactylifera*/Mean peak position of *P. crispum*) × 2201 Mb (=1C value of *P. crispum*)^63^.

### GWAS panel

Phenotyping for the GWAS was conducted on date palm trees located on two farms in the United Arab Emirates. The farms are located at The Date Palm Research Center in Hamriyah, Ras Al-Khaimah (*n* = 46) [latitude:25.60859749156817, longitude: 55.93000173568726] and in Al-Shuwaib, Al-Ain, Abu Dhabi (*n* = 111) [latitude: 24.771976901905425, longitude: 55.812156200408936]. The population consists primarily of female commercial varieties (*n* = 145). Males (*n* = 12) growing on the farms were also sequenced primarily for the purpose of mapping the sex determining locus.

Khalal stage fruit samples were collected from spring to fall in 2016, and either snap frozen on liquid nitrogen for RNA-sequencing or collected as fresh fruits for photography, scanning (see below) and characterization of other fruit traits. Tamar stage fruits from the same trees were collected in summer 2017 for sugar and organic acid profiling. Leaf samples were collected for DNA extraction and genome sequencing.

Genomic DNA was extracted from either leaf or fruit mesocarp/epicarp tissue using plant DNeasy mini kit (Qiagen, Venlo, Netherlands). DNA extraction columns, and libraries prepared using Illumina Nextera (San Diego, CA) kit. A 2 × 100 bp paired-end sequencing was conducted on an Illumina HiSeq 2500 sequencer with up to eight libraries per lane. Reads were demultiplexed and those passing Illumina quality control filters were processed with Trimmomatic^45^ (v. 0.36) to remove contaminating adapter sequences. For adapter removal, we used the adapter and Nextera transposase sequence database included with the Trimmomatic (v. 0.32) download with the following setting ILLUMINACLIP:〈adapter library〉:2:30:10 MINLEN:76 to retain only read pairs where both reads were 76 bps or longer following trimming.

Reads were aligned to the unmasked BC4 male assembly (primary contigs only) using bwa mem (v. 0.7.15-r1140 [http://bio-bwa.sourceforge.net]). The bwa mem aligner was run with the -M option to mark supplementary reads (0 × 800 bitwise flag) as secondary (0 × 100). Sample alignments were processed with FixMateInformation (Picard-tools v. 2.8.2; http://broadinstitute.github.io/picard) to ensure consistency in paired-read information, SamSort (Picard-tools v. 2.8.2) to coordinate-sort the alignments, MarkDuplicates (Picard-tools v. 2.8.2) to flag duplicate read pairs, and with GATK^64^ IndelRealignerTargetCreator/IndelRealigner tool (GATK v. 3.7-0) to realign reads in indel regions. Sample alignments were validated at each step using ValidateSam (Picard-tools v. 2.8.2) to ensure no errors in production. Processed alignments were summarized with CollectAlignmentSummaryMetrics (Picard-tools v. 2.8.2) and Samtools [https://github.com/samtools/samtools].

### SNP calling and genotyping

SNP-calling and genotyping was performed with the GATK (v. 3.7-0) HaplotypeCaller run in GVCF mode followed by joint-genotyping with GenotypeGVCFs [https://software.broadinstitute.org/gatk/]. Reads were filtered from the HaplotypeCaller step to exclude those with a mapping quality less than 20 and to exclude those marked as polymerase chain reaction (PCR) duplicates or secondary alignments (see above). This approach yielded 32,384,028 SNPs across all samples. SNP filtering was conducted by applying hard filters to the raw variants using GATK v. 4.0.2.1. We filtered the raw call set to exclude SNPs with low (<785) and high depth (>2862) summed across samples. We also excluded multi-allelic SNPs, SNPs within 10 bp of indel polymorphisms, and SNPs meeting the following conditions: QUAL < 30 and QD < 5.0. Genotypes were set as missing if DP was below 5 or above 20, as well as SNPs with a genotype call rate < 80%, or a minor allele frequency below 0.01. We estimated a *P* value for each site from a Hardy–Weinberg Equilibrium test using VCFtools^65^ and filtered out SNPs showing an excess in heterozygosity (exact test, *P* < 0.05). This procedure yielded a filtered call set of 7,149,205 SNPs.

### Statistical analysis

All statistical analysis was conducted in the R statistical computing language unless otherwise indicated.

### LD analysis

LD was estimated using a method for estimating *r*^2^ that is appropriate for unphased data (see VCFtools^65^). The LD decay curve for the GWAS panel was calculated as in Flowers et al.^4^. Briefly, *r*^2^ was calculated for unphased SNPs with minor allele frequency greater than 10% using the–geno-ld option in VCFtools (v. 0.1.14). Decay curves were generated by fitting a curve to the pairwise *r*^2^ estimates by physical distance between SNP pairs with nonlinear least squares using an approach adapted from Marroni et al.^66^. The half-decay distance was then calculated as the distance at which *r*^2^ is half its maximum value (i.e., 1 bp distance).

### Characterization of fruit color

Eight khalal stage fruits free of injury per date palm variety were harvested, rinsed with tap water to remove any dust and then air-dried. The fruits were sliced longitudinally, and fruit color was then measured using two strategies. First, we photographed the sliced fruits with a color checker in a camera photo studio box, where the pictures were taken on a white background with a digital camera. The color of the fruit was analyzed with ImageJ software^67^ using the RGB color parameters.

Second, we used a complementary approach, where we used Tomato Analyzer software^68^ v.2.2 to obtain estimates of color parameters L*, a*, b*. The L* coordinate expresses the darkness and the lightness of the color and ranges from black (0) to white (100). Coordinates a* and b* express color direction, where +a* is in the red direction, −a* in the green direction, +b* in the yellow direction and −b* in the blue direction^68^. Image acquisition and analysis was done as described in Rodríguez et al.^27^. Sliced fruits were place on a scanner with a black background and covered to avoid the effects of ambient light. Scanned pictures were saved as JPEG files and the estimates of color parameters L*, a*, b* were done on each fruit. The average of all fruit was calculated. The two methods were highly correlated, so we used color index a*/b* in order to evaluate the differences in skin colors of the fruits and used that for the association study.

### Fruit anthocyanin content

Total anthocyanin was extracted from three replicates of khalal stage fruit from each date palm variety using fruits snap-frozen on liquid nitrogen following the procedure described in Rabino and Mancinelli^69^ with minor modification. Briefly, anthocyanin from frozen fruit skin (100 mg) was ground into fine powder and extracted in 1 ml of acidic methanol (1% HCl) by incubation at room temperature in the dark for 18 h, followed by centrifugation for 10 min at 12,000 *g*. Quantification of total anthocyanin was done using the absorbance measured by a spectrophotometer using the equation

Total anthocyanin = (A530-0.25 × A657)/FW, where A530 and A657 nm are the absorbance and FW is the wet weight of the plant material (g).

### Fruit size

Fruit photographs used for color analysis (see above) included a ruler as a size standard. ImageJ^67^ (v. 2) and Tomato analyzer software^27^ were then used to estimate fruit length and width.

### Fruit sugar and acid content

Fruit sucrose, glucose, and fructose were quantified from 125 varieties at the tamar stage when fruits are dry, ripening is complete and the stage at which dates are typically consumed. Fruits were snap-frozen at −20 °C and between 10 and 15 fruits per variety were immediately maintained at −20 °C through arrival at Montpellier (French Agricultural Research Centre for International Development, CIRAD) where high performance liquid chromatography analysis was performed. A single measurement from two pooled fruits was obtained for each of the sugar and acid traits. Date pieces (without the stone) were frozen with liquid nitrogen and ground in powder, put in two separate tight glass vials, stored at −20 °C until sampling. For the dry matter, in duplicate, 1 g of sample was weighed and placed in a stove under vacuum at 70 °C for 72 h. A control was checked for 4 days to determine the optimum duration. Sugar extractions were performed using the method adapted from Bchir et al.^70^. For each sample, 500 mg date paste and 10 ml of 80% ethanol were placed in a 15 ml tube, heated for 5 min at 80 °C in a water bath. Each tube was then agitated at first manually and then mechanically for 15 min for better spreading. After centrifugation at 9000 × *g* (Avanti J-E centrifuge; Beckman-Coulter, Brea, CA, USA), the bottom was extracted twice and the supernatants gathered, filtered at 0.45 µm and injected. The method was tested with acidic water (0.01 N H_2_SO_4_). Sample standards were Sigma-Aldrich (St. Louis, MO, USA) were used.

### Fruit moisture content

Fruit sampling was performed as in fruit sugar and acid content section above. Date pulp from two fruits was recovered and ground with liquid nitrogen to homogenize sample and stored at −80 °C to obtain a single measurement per variety. The moisture content was gravimetrically determined by measuring the weight loss of 2.5 g of date pulp samples, dried at 70 °C until the samples reached a stable weight.

### Genome-wide association analysis

We ran the genome-wide association mapping analysis using the Gapit R package^25^. For computational efficiency and to minimize multiple-testing issues but provide dense coverage with respect to the LD decay distance, we used a 5.5% downsampled random SNP set (392,948 SNPs). A CMLM^26^ using both population structure and kinship information as covariates was performed on the genotypes from the 157 date palm samples. Population structure was inferred with a principal component analysis (PCA) generated by Gapit using 1% of the SNPs (randomly sampled). Gapit further used the first five components of the PCA (Fig. 1a; Supplementary Data 2). Kinship was inferred using the VanRaden algorithm (Supplementary Data 3). Significant SNPs were identified using a conservative Bonferroni threshold of *P* < 1.27 × 10^−7^. For traits with significant results, we further performed a second GWAS analysis using the full SNP set on particular LGs where significant SNPs were identified.

### Characterization of *Ibn Majid* and the *VIR* gene

We previously identified a *copia*-like retrotransposon insertion polymorphism in exon 3 of an R2R3-MYB transcription factor^13^ (NCBI Gene ID: LOC103717680) that is orthologous to the *Virescens (VIR)* gene in oil palm^28^. To characterize this retrotransposon, we PCR-amplified the element long terminal repeats (as well as adjacent *VIR* gene sequence) in Thory and Empress varieties collected from the USDA farm in Thermal, California and the USDA/UC Riverside farm respectively, using GoTaq PCR Core Systems (Promega, Madison, WI USA) buffer and polymerase.

The primer pairs 5′-TGT GTC CGG CAT TGC ACT TCT-3′ (forward) and 5′-GCT CAA TGT TGA TGT TCT TGT TGG-3′ (reverse) were used for the 5′ LTR, and 5′-ACTC TGA CTA CCA AGT ACT TGA TG-3′ (forward) and 5′-CTG CAC TAT TAT CAC AGT AGA TGG-3′ (reverse) for the 3′ LTR. Amplified products were sent for Sanger sequencing at GeneWiz (South Plainfield, New Jersey). Our genome assembly also contains a complete copy of the insertion (~11.7 kb). BLAST was used to align the insertion against itself in order to identify the matching long terminal repeat regions. The program LTRdigest^71^ was used to confirm the BLAST results. A BLAST search queried the full *Ibn Majid* sequence against the date palm genome to determine copy number.

Supplementary Table 11 provides coordinates of our manual annotation of the *VIR* gene in the BC4 male assembly. Genotyping of the *Ibn Majid* insertion in *VIR* exon 3 in date palm varieties was performed by manual inspection of aligned reads spanning the insertion region in JBrowse^72^. Since the BC4 male genome assembly has the insertion allele (*VIR*^IM^, see Fig. 3), mapped reads originating from wild type (*VIR*^*+*^), or non-insertion alleles, are soft-clipped at the exon 3-insertion boundary. We scored the presence of soft-clipped reads (supporting the presence of a *VIR*^+^ allele) or unclipped reads spanning the exon 3-insertion boundary (supporting the presence of a *VIR*^IM^ insertion allele) to identify genotypes. We repeated this procedure by examining read alignments at both the 5′ and 3′ ends of the insertion in the BC4 male assembly and samples where both 5′ and 3′ genotypes yielded matching genotypes were retained for analysis. Given our interest in fruit color phenotypes, we genotyped female palms only.

### Characterization of invertases and deletion polymorphisms

Examination of genes in the sugar composition QTL on LG 14 (Supplementary Data 6) initially revealed three positional candidates—an alkaline/neutral invertase (chr14G0028200) and two adjacent cell wall invertases (chr14G0022900 and chr14G0023100) predicted by our gene annotation pipeline. We checked for potential additional unannotated copies of invertase in this region by aligning predicted transcripts for each of the three genes to this region using the Splign transcript to genomic alignment tool^73^. This recovered a minus strand sequence (which we refer to as *CWINV2*), with close homology to the flanking invertases *CWINV1* and *CWINV3* at 2,489,373 to 2,485,592, but multiple insertion/deletions in regions homologous to invertase CDS exons.

Coverage depth for deletion variation analysis was determined in 500 bp non-overlapping bins with samtools bedcov^74^ (v. 1.9) using default settings. Raw depth values were normalized independently for each sample by dividing the raw depth of each bin by the median raw depth of all bins on LG 14 following by log_2_ transformation following Flowers et al.^75^. Samples were genotyped into homozygous deletion and alternate genotype classes for the 40 kb deletion by manual inspection of Supplementary Fig. 12. Homozygous genotypes for the deletion upstream of *A/N-INV1* (Fig. 4, Supplementary Fig. 13) were called by setting a threshold requiring that at least one 500 bp interval in the 5 kb deletion region have log_2_ normalized depth less than −5. At present, it is not possible to distinguish heterozygotes for deletion alleles from insertion homozygotes owing to the moderate coverage in our re-sequencing data.

### Invertase enzyme assay

Two sucrose and two reducing sugar varieties were chosen for the invertase assay. The experiment was conducted on two days with all four varieties represented by a single fruit on each day. Assays were conducted on one khalal stage fruit snap-frozen at the time of collection (see above) followed by storage at −80 °C. Crude extracts were obtained from the frozen date fruit following the protocol of Hasegawa and Smolensky^33^. Each frozen fruit was pulverized with mortar and pestle (with seed removed), and then ground in a kitchen blender, and 5 g placed in cold extraction buffer (20 ml 4.0% NaCl, 1 g polyvinylpyrrolidone, PVP). An additional maceration step was conducted in a laboratory homogenizer for 1–2 min. The extract was then centrifuged at 20,000 × *g* for 15 min at 4 °C. The supernatant containing soluble invertase was stored on ice and the remainder centrifuged a second time at 20,000 × *g* for 15 min at 4 °C. The supernatants were combined and 10 ml dialyzed against cold water at 4° overnight to remove sugars from the extract. The sample was then split, and one-half of the sample boiled at 100 °C to measure background activity from potential contaminating sugar from the fruit. Invertase activity of unboiled and boiled crude extracts was then measured by colorimetric assay on a Synergy H1 microplate reader with a coupled enzyme assay kit (Sigma catalog no. MAK118) following the manufacturer’s instructions.

### Fruit RNA-Seq analysis

Two RNA-Seq datasets were collected to address questions about fruit development and variation in fruit traits. RNA-Seq at different fruit development stages was conducted on fruits collected in 2014 from replicate trees located on the grounds of the United Arab Emirates University, Date Palm Tissue Culture Laboratory in Al-Ain, UAE. For this experiment, three or four separate trees of Khenezi (a variety with red fruit) and Khalas (yellow fruit) varieties were sampled repeatedly at 45, 75, 105, 120, and 135 days post pollination and fruits snap-frozen on liquid nitrogen. RNA was extracted from a single fruit from each three or more trees per variety following standard protocols for TruSeq library preparation, and 2 × 101 bp paired-end sequencing performed on an Illumina HiSeq 2500.

A second experiment was conducted on khalal stage fruit collected at the Al-Shuwaib farm in 2016. Three fruits were collected from each of eight palms each of a different variety chosen based on their being at or close to the extremes of the sucrose and reducing sugar type distributions (i.e., high and low sucrose concentration). Fruits were processed as described above and libraries constructed with Nextera library preparation kit (Illumina) and 2 × 76 bp paired-end sequencing performed on a NextSeq (Illumina) instrument.

Differential expression analysis was performed by trimming raw sequencing reads with Trimmomatic^45^ (v 0.36) with parameters ILLUMINACLIP:〈adapter fasta〉:2:30:10 TRAILING:3 LEADING:3 SLIDINGWINDOW:4:15 MINLEN:36. Reads were then aligned to the BC4 male reference genome with the STAR split read aligner^47^ (v. 2.5.3a) and read counts generated per gene by taking the union of exons with htseq-count^76^ (v. 0.9.1) set to include only uniquely mapped reads (i.e., htseq-count options --type = exon--mode = union--nonunique = none). Read count normalization was conducted with the median-of-ratios method of DESeq2^77^ (v. 1.8.2). Tests of differential expression of *Virescens* (Pdac_HC_chr4G0137100) between red (Khenezi, *n* = 3 replicate libraries) and yellow (Khalas, *n* = 3 or 4 replicate libraries) varieties were conducted separately for each of the fruit development time points of 45, 75, 105, 120, and 135 days post pollination. *P* values are reported for a Wald’s test of the hypothesis of no fold-difference between Khenezi and Khalas expression at each stage.

RNA-seq analysis of differential gene expression of invertases *A/N-INV1, CWINV1*, and *CWINV3* (Pdac_HC_chr14G0028200, Pdac_HC_chr14G0022900, and Pdac_HC_chr14G0023100, respectively) between sucrose (*n* = 4 varieties) and reducing-sugar types (*n* = 4 varieties) was conducted by building three libraries per variety from RNA extracted independently from three different fruits followed by sequencing each library. Analysis of differential expression between sucrose-type and reducing-type varieties was then performed by aligning reads with STAR (see above), counting reads with htseq-count, and generating raw count matrices in DESeq2. Raw counts per gene were then summed across libraries for each variety owing to low read counts in some libraries. Subsequent analysis was conducted by first dropping low count genes (genes with <10 reads summed across all 8 samples) followed by the standard DESeq2 (v. 1.22.2) work flow with four biological replicates (i.e., date palm varieties) in each treatment group. Uncorrected *P* values for the hypothesis of no differential expression are presented in the main text for three candidate genes.

### Reporting summary

Further information on research design is available in the Nature Research Reporting Summary linked to this article.

## Supplementary information

Supplementary Information

Peer Review

Reporting summary

Description of Additional Supplementary Files

Supplementary Dataset 1

Supplementary Dataset 2

Supplementary Dataset 3

Supplementary Dataset 4

Supplementary Dataset 5

Supplementary Dataset 6

Source Data file

## Data Availability

Data supporting the findings of this work are available within the paper and its Supplementary Information files. A reporting summary for this Article is available as a Supplementary Information file. The datasets generated and analyzed during the current study are available from the corresponding author upon request. The genome and various accessory files have been deposited in the Sequence Read Archive (SRA) under NCBI BioProject PRJNA322046 [https://www.ncbi.nlm.nih.gov/bioproject/322046]. Short read sequencing data from the GWAS panel have been deposited in the SRA under PRJNA505141. RNA-seq data for differential gene expression experiments have been deposited in the SRA under PRJNA505138. A genome browser was constructed using Tripal^78^ (v2.1) and can be explored visually at [https://datepalmgenomehub.abudhabi.nyu.edu]. SNP data for the GWAS have been deposited at the Dryad Digital Repository [10.5061/dryad.3mc4265]. The source data underlying Figs. 1b–g, 2, 3b, c, e–g, 4, and 5, Supplementary Figs. 1–13, and Supplementary Tables 12–14 are provided as a Source Data file.
