# Supplementary material for: Genome-wide association mapping of date palm fruit traits
Source: Nat Commun. 2019 Oct 15;10:4680. doi: 10.1038/s41467-019-12604-9 (PMC6794320; doi:10.1038/s41467-019-12604-9)
Supplement: Supplementary file 1 — Supplementary Information [file 41467_2019_12604_MOESM1_ESM.pdf]

Genome-wide association mapping of date palm fruit traits

Hazzouri *et al.*

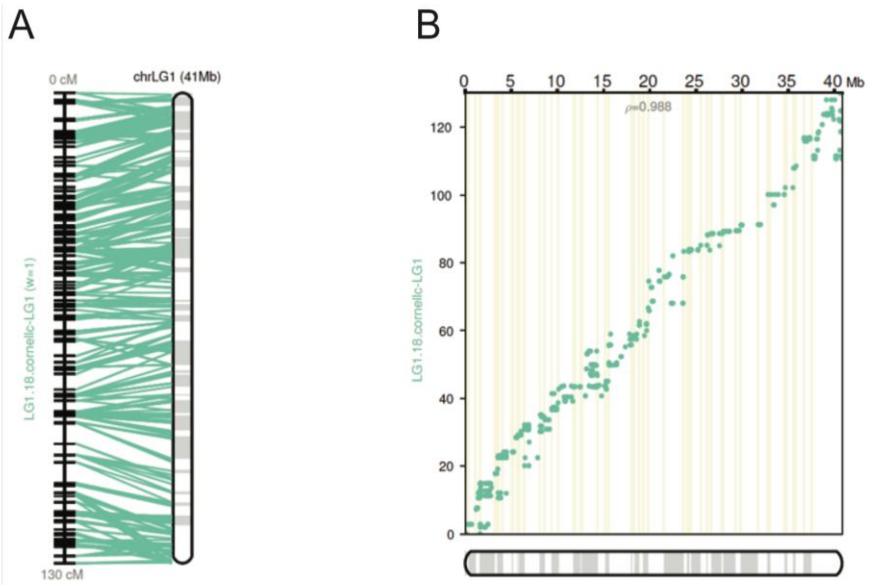

Supplementary Fig. 1. Anchoring of BC4 male contigs to linkage groups with ALLMAPS.

(A) The markers placed on the genetic map (ticks on left) are connected (green lines) to their position on the linkage group 1 ideogram. (B) Relationship between physical and genetic positions on linkage group 1. Source data are provided as a Source Data file.

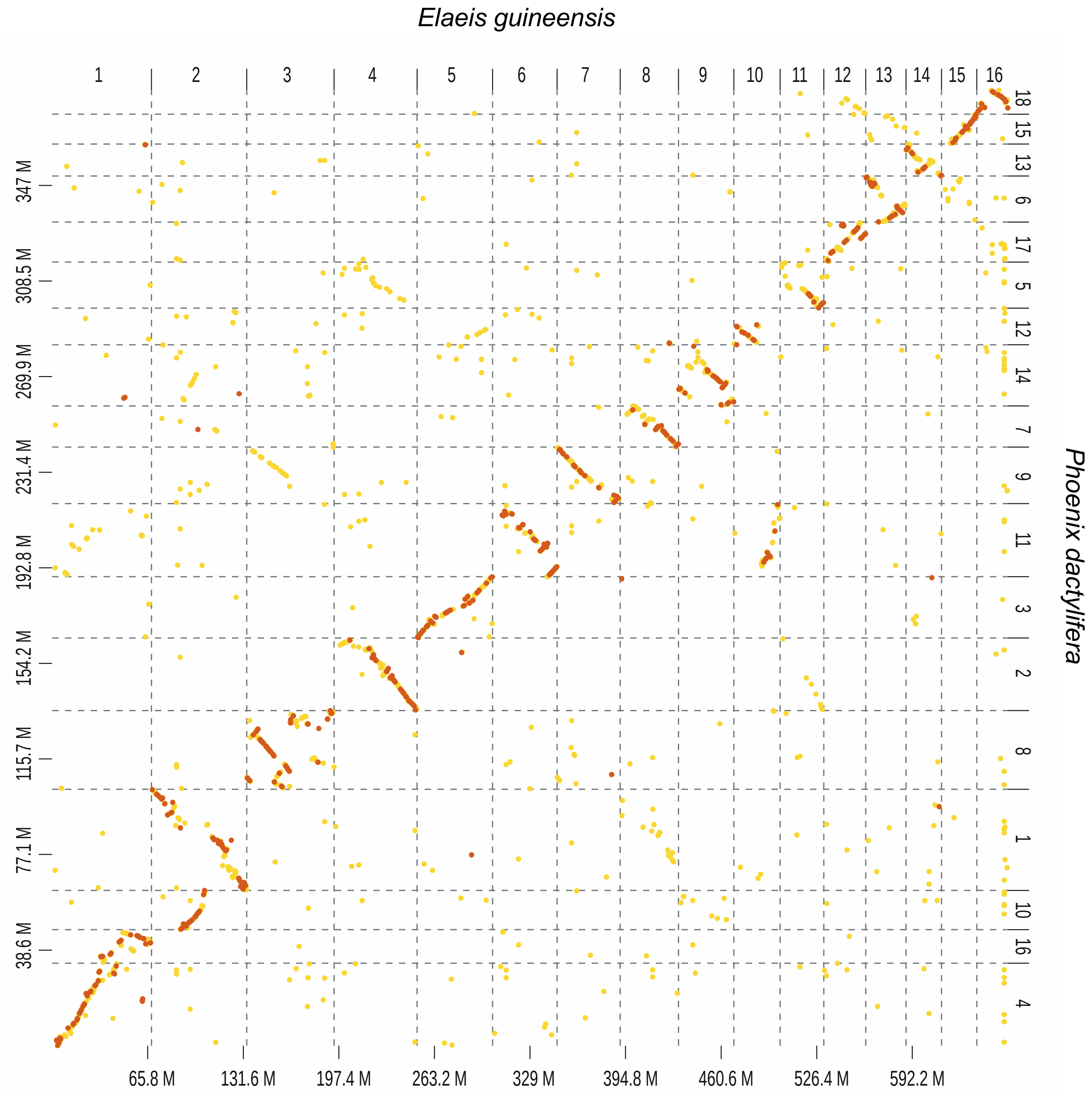

Supplementary Fig. 2. Syntenic dotplot of the BC4 male and oil palm genomes. Bc4 male: 2n=36; oil palm: 2n=32. Source data are provided as a Source Data file.

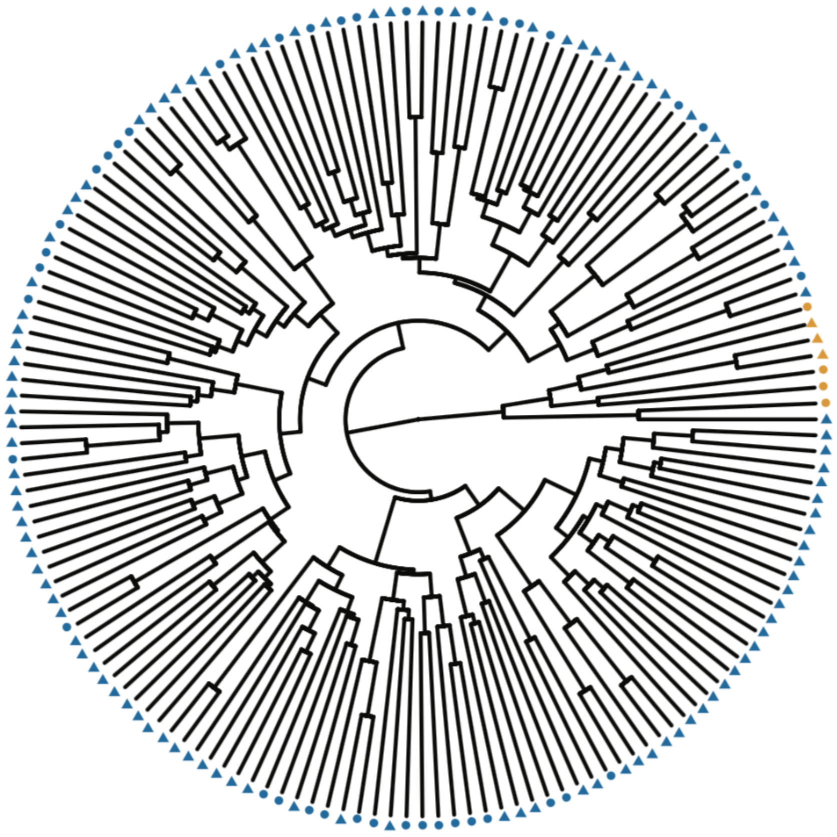

Supplementary Fig. 3. Hierarchical clustering of 157 date palms in the mapping population based on kinship coefficients.

Kinship coefficients were calculated according to the VanRaden method as implemented in Gapit software[1,2]. Clustering diagram was produced with the *hclust* function in *R*[3] with Euclidean distances as input. Triangles indicate trees sampled from the Al-Shuwaib farm, Al Ain, Abu Dhabi emirate, while dots indicate trees sampled from al Hamriyah farm in the Ras al Khaimah emirate. Blue and orange indicate cultivars clustering in Middle East or North African clades, respectively. Source data are provided as a Source Data file.

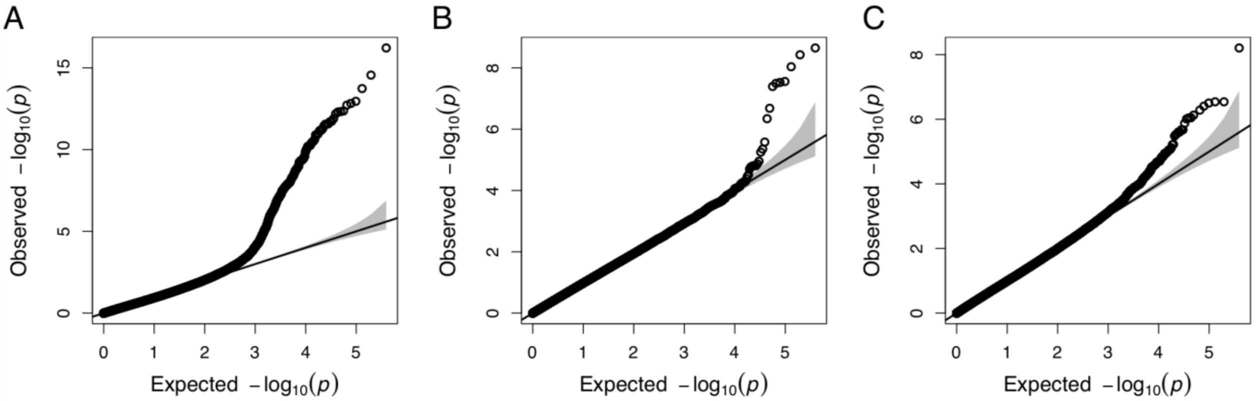

Supplementary Fig 4. Quantile-quantile plots for GWAS mapping.

(A) Sex (inflation factor = 0.932), (B) fruit color as assessed using the a/b (inflation factor = 0.990), and (C) sugar content variation assessed by [sucrose/(sucrose + glucose + fructose)] (inflation factor = 1.038). These plots correspond to the mapping using the downsampled dataset. Corresponding Manhattan plots are presented in Figs. 2, 3 and 5 in the main text. Source data are provided as a Source Data file.

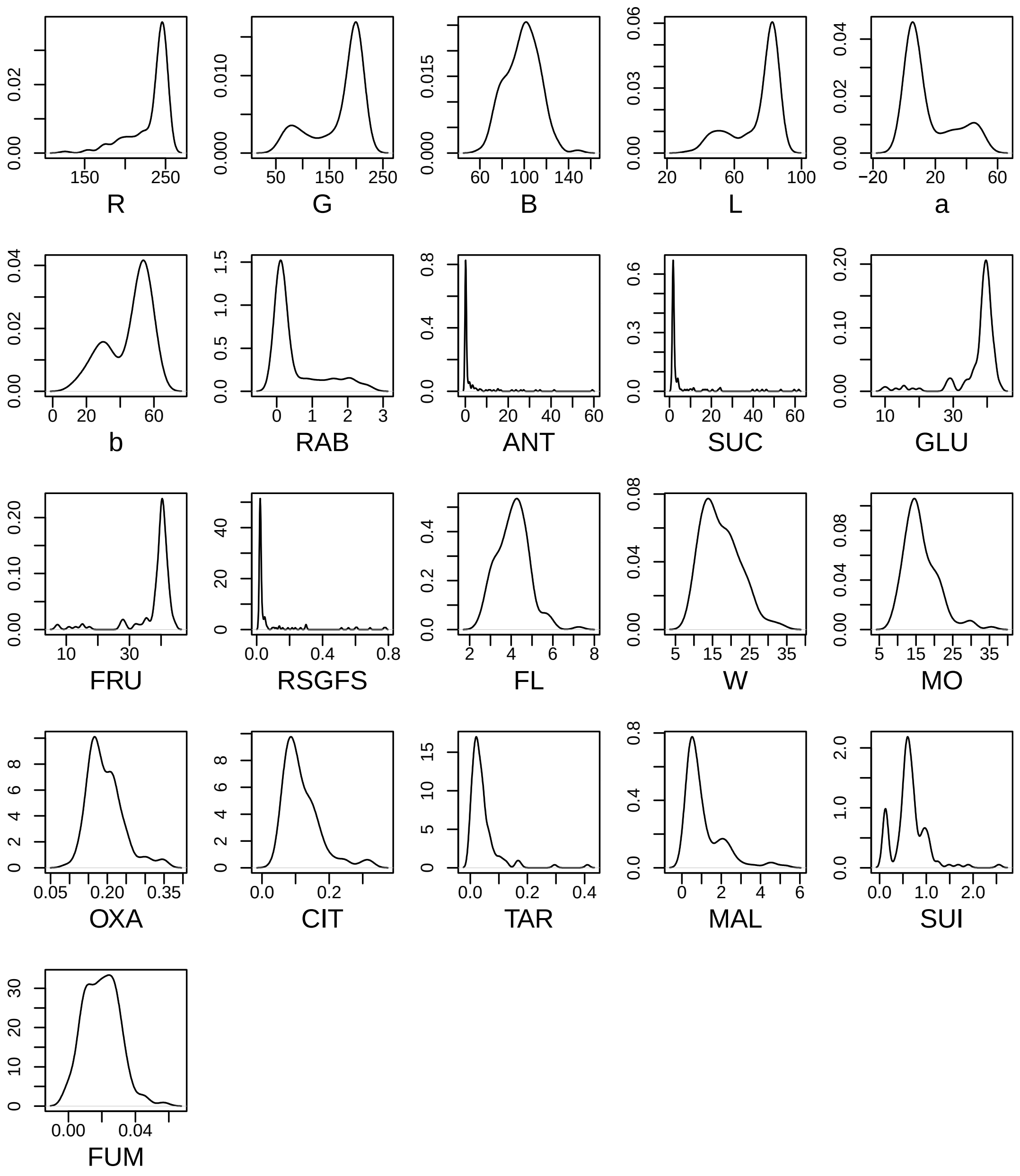

Supplementary Fig. 5. Density distribution of each phenotype measured on 145 female date palms.

Fruit color was described by different parameters: R, G and B are color parameters of the RGB color model while L, a and b are parameters value from the CIELAB color space. RAB = ratio a/b. ANT = level of anthocyanin. SUC, GLU and FRU are the levels of sucrose, glucose and fructose (g/100g of dry matter), respectively while RSGFS is the ratio of sucrose over total sugar [sucrose/(sucrose+glucose+fructose)]. FL = fruit length, W = Fruit weight, MO = Fruit moisture. OXA, CIT, TAR, MAL, SUI, FUM are level of oxalic, citric, tartaric, malic, succinic, fumaric acids, respectively (g/100g). Source data are provided as a Source Data file.

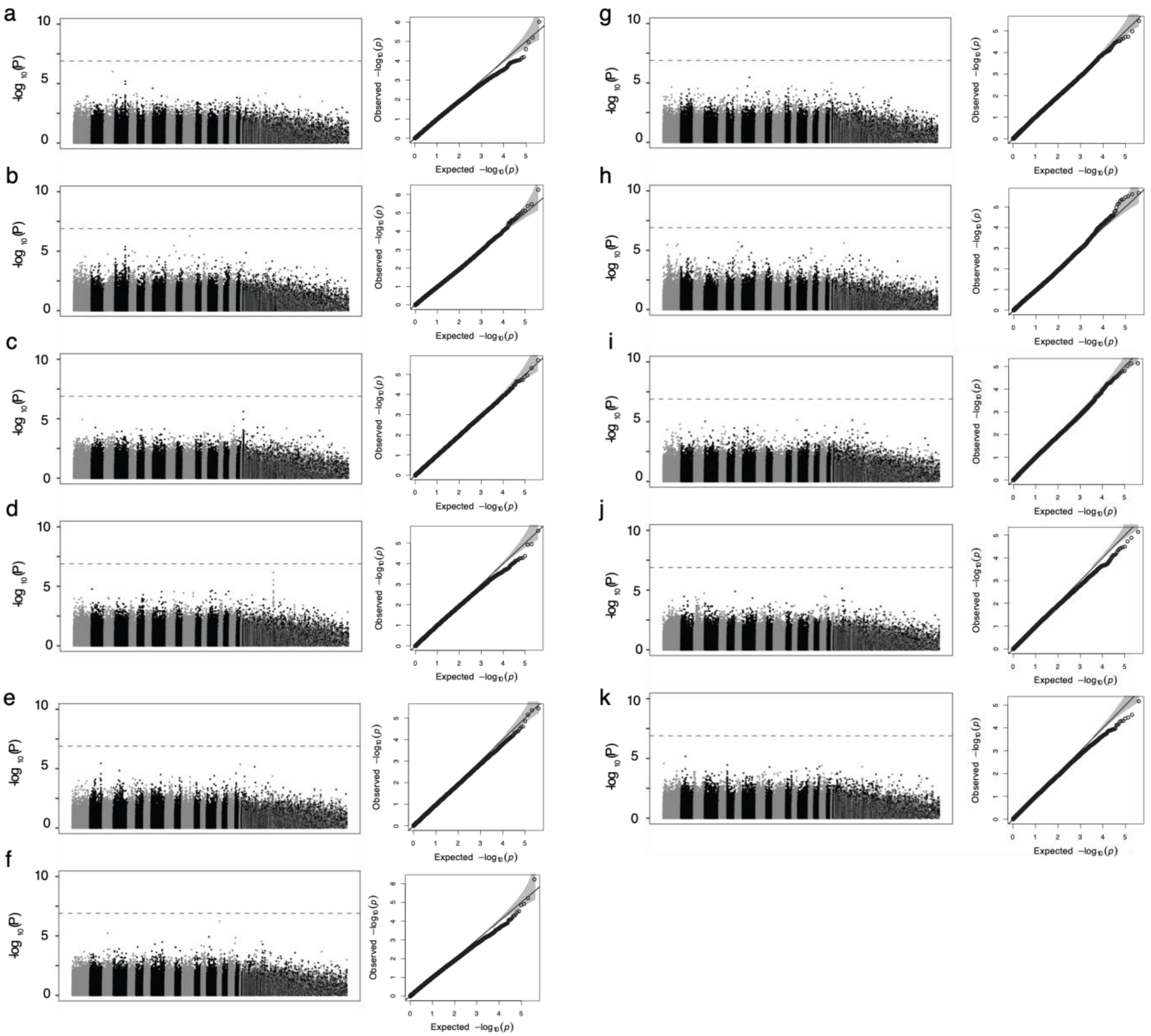

Supplementary Fig. 6. Genome-wide association studies for various traits without significant associations.

These were based on a Bonferroni threshold of 5% ($P < 1.3 \times 10^{-8}$). Left: Manhattan plot with Bonferroni threshold shown as a dotted line, right: QQ plot. (A) color parameter B (RGB system), (B) Anthocyanin level, (C) Fruit length, (D) Fruit weight, (E) Moisture, (F) Oxalic acid, (G) Citric acid, (H) Tartaric acid, (I) Malic acid, (J) Succinic acid, and (K) Fumaric acid. Source data are provided as a Source Data file.

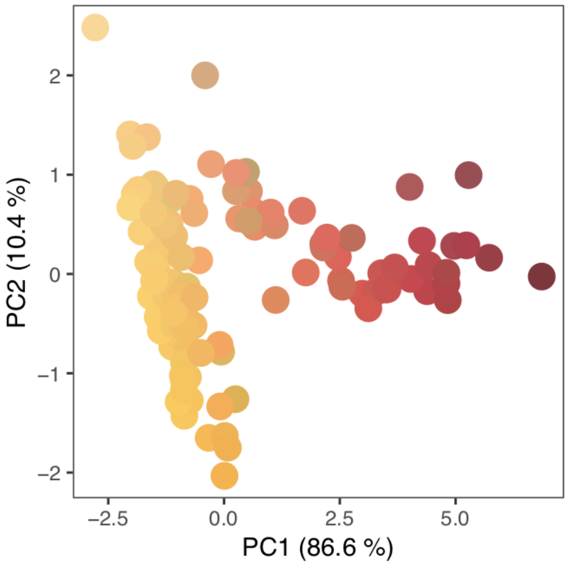

Supplementary Fig. 7. Principal Component Analysis on fruit color traits in 145 date palms.

Principal component analysis (PCA) was performed on color parameters (R, G, B, L, a, b, a/b) and anthocyanin levels. Dots are colored according to the actual fruit color as inferred by R, G, and B. Source data are provided as a Source Data file.

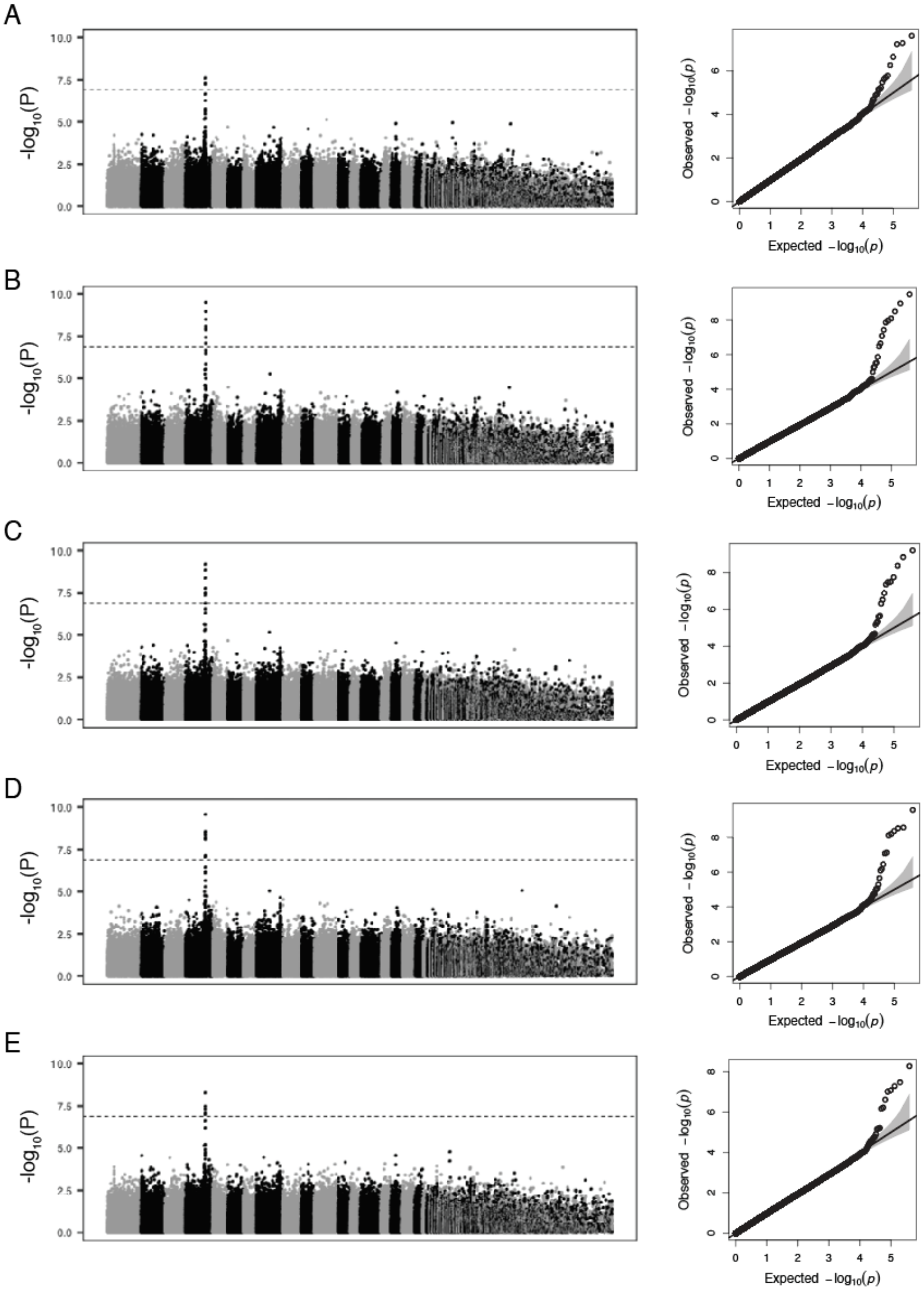

Supplementary Fig. 8. Genome-wide association studies of fruit color with significant associations.

These were based on using a Bonferroni threshold of 5% ($P < 1.3 \times 10^{-8}$). Left: Manhattan plot with Bonferroni threshold shown as a dotted line; right: Quantile-Quantile plot. (A) R (RGB system), (B): G (RGB system), (C) L (CIELAB color space), (D) a (CIELAB color space), (E) b (CIELAB color space). Source data are provided as a Source Data file.

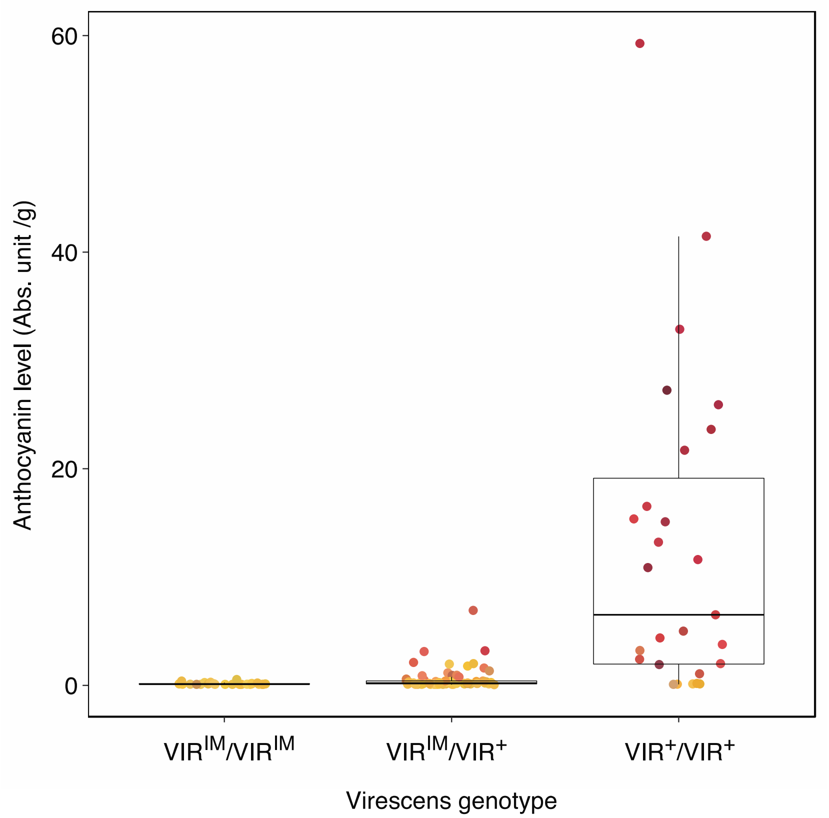

Supplementary Fig. 9. Boxplot distributions of fruit anthocyanin level by *Ibn Majid* genotype.

Center line is the median, bounds of box represent the first and third quartiles, the upper and lower whiskers extend from the hinge to the largest or smallest value, respectively, no further than 1.5 * IQR from the hinge (where IQR is the inter-quartile range, or distance between the first and third quartiles). Points are colored as described in the Fig. 3 legend. Source data are provided as a Source Data file.

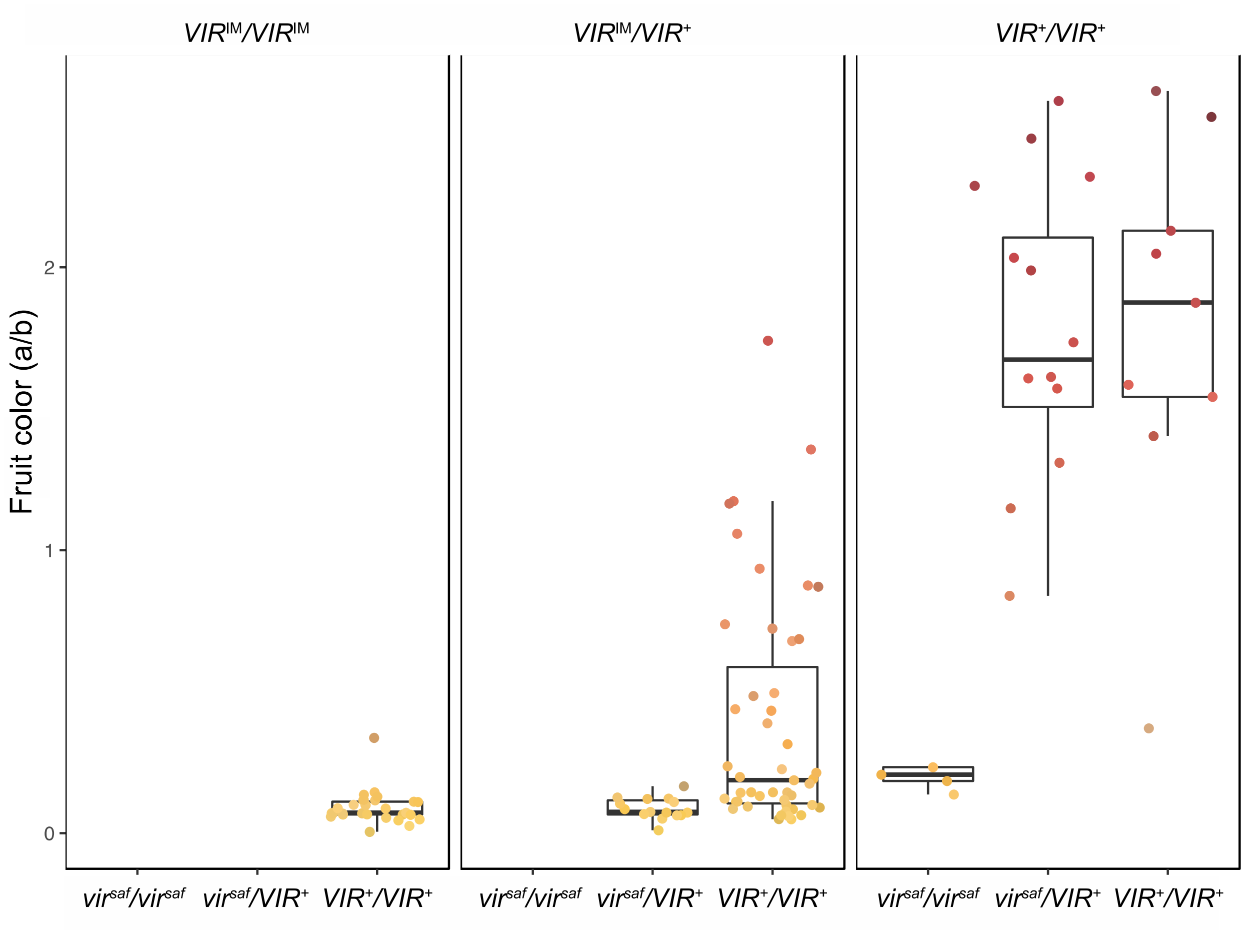

Supplementary Fig. 10. Fruit color variation by genotype at candidate functional mutations in *Virescens*.

Center line is the median, bounds of box represent the first and third quartiles, the upper and lower whiskers extend from the hinge to the largest or smallest value, respectively, no further than 1.5 * IQR from the hinge (where IQR is the inter-quartile range, or distance between the first and third quartiles). Points are colored as described in the Fig. 3 legend. Each panel represents a *Virescens* genotype at the *Ibn Majid* retrotransposon insertion site. Each column within panels represents a *Virescens* genotype at the translation start codon site. See main text for additional details concerning allele nomenclature. Source data are provided as a Source Data file.

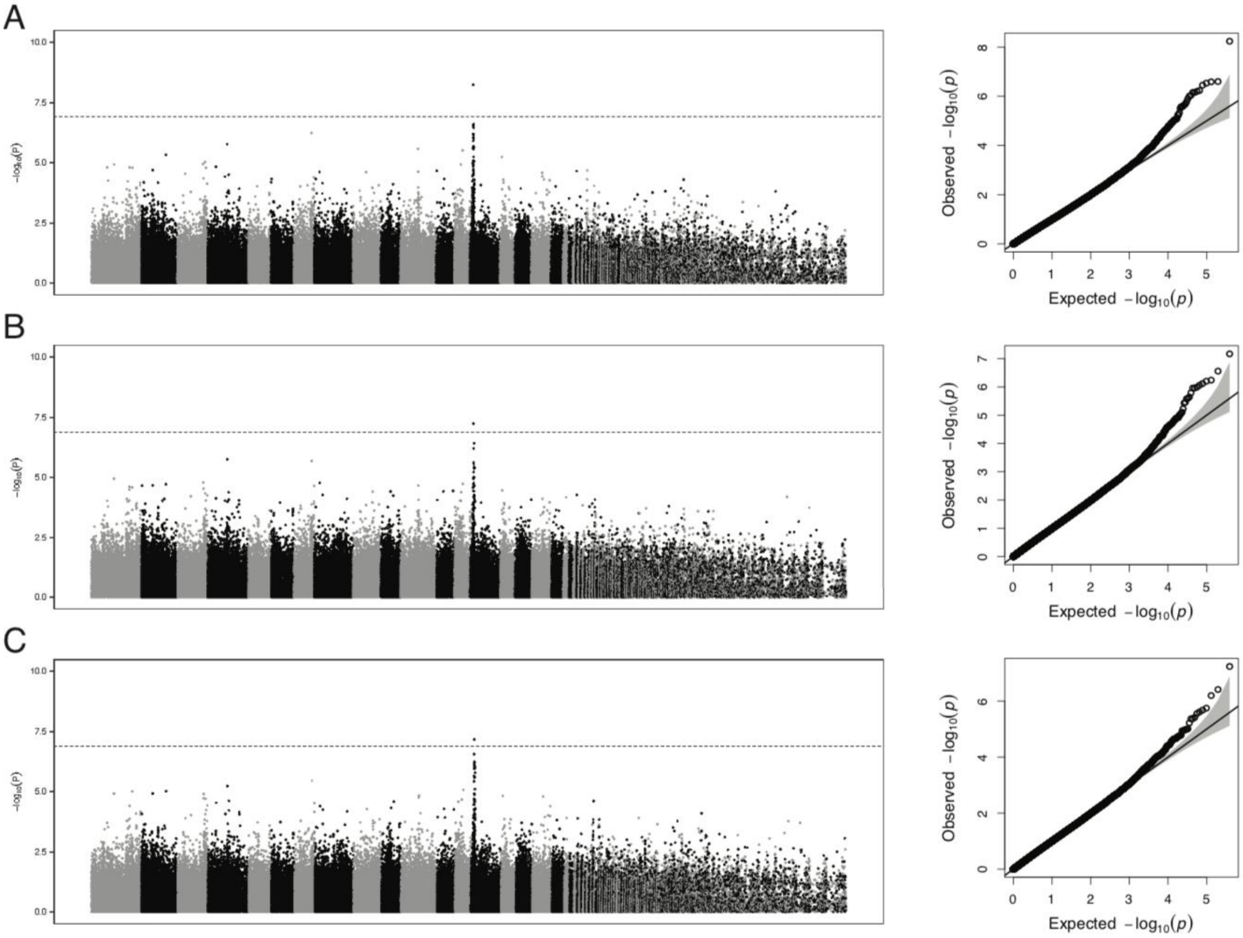

Supplementary Fig. 11. Genome-wide association studies on the downsampled SNP set for sugar-related traits with significant associations.

These were based on using a Bonferroni threshold of 5% ($P < 1.3 \times 10^{-8}$). Left: Manhattan plot with Bonferroni threshold shown as a dotted line; right: Quantile-Quantile plot. A: sucrose, B: glucose and C: fructose. Source data are provided as a Source Data file.

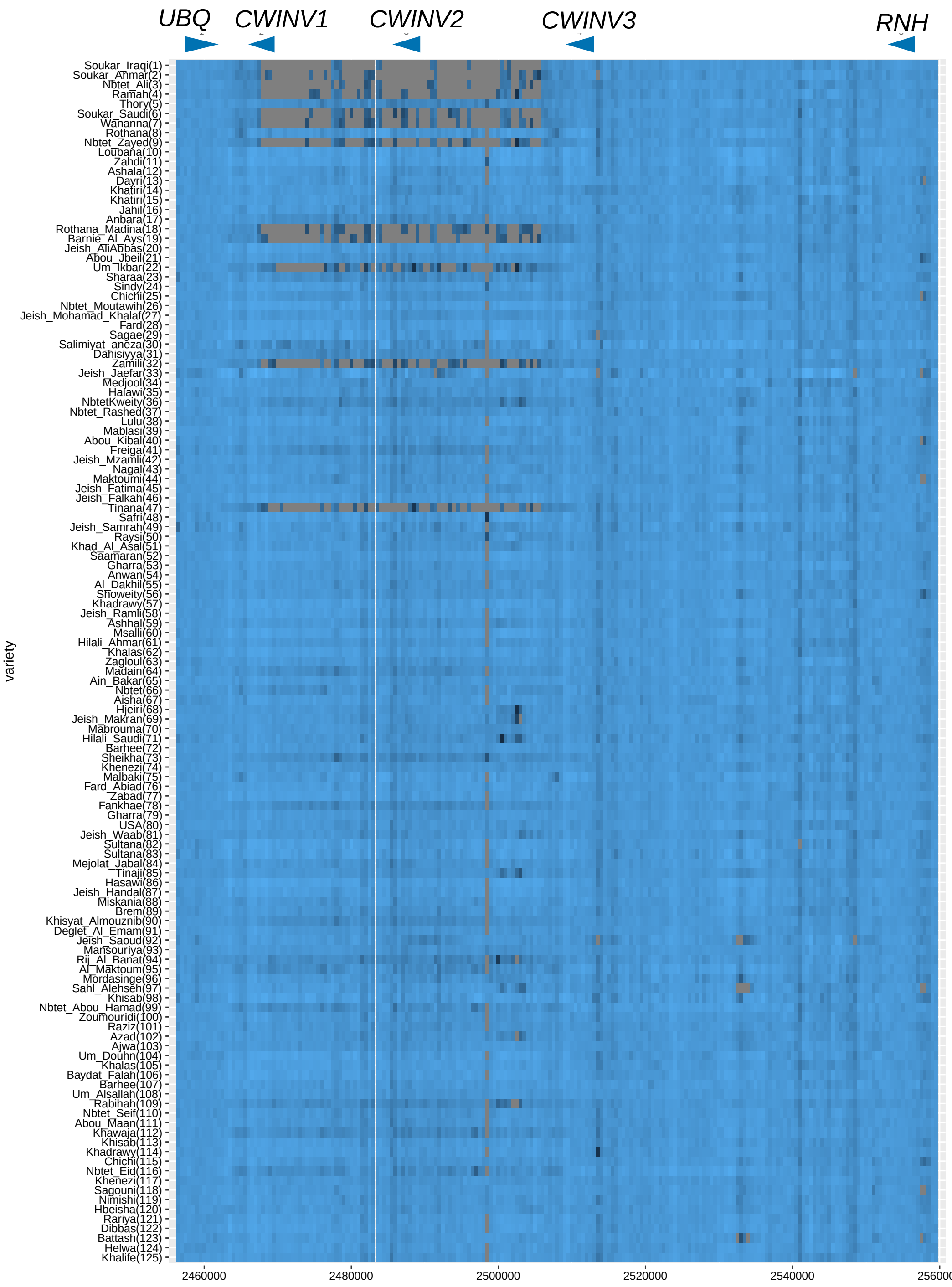

Supplementary Fig. 12. Normalized coverage depth in the 40 kb deletion region on LG 14 for all samples with sugar measurements from tamar stage fruit.

See also Fig. 5. All gene models are shown in the region including an un-annotated sequence (*CWINV2*, see main text). Gene abbreviations are as in Fig. 5. Numbers in parenthesis next to variety names are the sucrose to total sugar rank among the 125 varieties with sugar measurements, where (1) is highest sucrose. Colors are as described in Fig. 5 legend. Source data are provided as a Source Data file

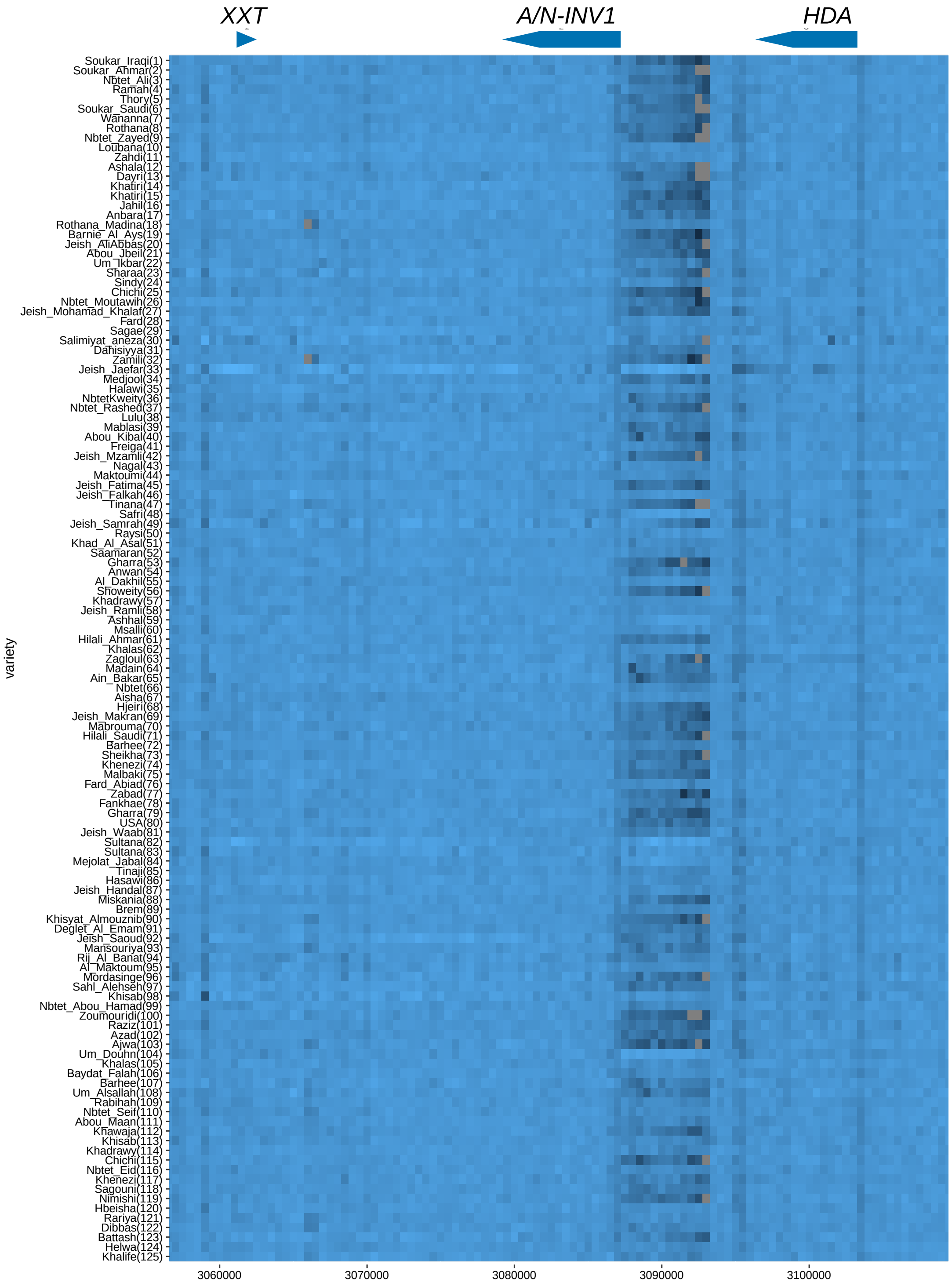

Supplementary Fig. 13. Normalized coverage depth in the 5 kb deletion region on LG 14.

These are for all samples with sugar measurements from tamar stage fruit (see also Fig. 5). All gene models are shown in the region. Gene abbreviations are as in Fig. 5. Numbers in parenthesis next to variety names are the sucrose to total sugar rank among the 125 varieties with sugar measurements, where (1) is highest sucrose. Colors are as described in Fig. 5 legend. Source data are provided as a Source Data file.

Supplementary Table 1. Summary of the BC4 male date palm genome assembly.

	Total reads (million)		Total sequence (Gb)	Coverage	N50 (kb)
**PacBio long reads**	6.4		72	92.9X	18.5
**Illumina short insert**	110		10.3	13.8X	-
	**Total**	**N50 (kb)**	**Max Size (Mb)**	**Anchored (Mb)**	**Unplaced (Mb)**
**Primary contigs**	2,706	897.2	12.1	385.6	386.7
**Haplotigs**	9,753	70.9	2.2	-	547.4
	**LGs**	**Total Size (Mb)**	**Genes**	**GC (%)**	**BUSCOs**
**Final assembly**	18	772.3	28,595	40.3	92.5%

Supplementary Table 2. BC4 male genome assembly summary statistic from Falcon-UNZIP.

	Primary assembly	Haplotigs
**Contigs**	2706	9753
**Total size (Mb)**	772.3	547.4
**Longest (Mb)**	12.1	2.2
**Shortest (kb)**	8.3	5.1
**N50 (kb)**	897.2	70.9
**L50**	196	1385

Supplementary Table 3. Percent mapped RNA-Seq reads of different tissues to the BC4 genome.

Tissue	Total reads	Mapped reads	% mapped reads
**Root**	17,588,072	17,425,946	99.08
**Fruit**	53,589,875	53,323,931	99.50
**Leaf**	25,907,873	25,834,666	99.72
**Pollen**	129,870,717	128,696,250	99.10
**Flower**	115,766,623	114,067,198	98.53

Supplementary Table 4. Summary of genome assembly completeness[a].

	Primary assembly	Haplotigs	Combined
**Complete single copy**	1089 (75.6%)	735(50.8%)	577 (40.1%)
**Complete duplicated**	230 (16.0%)	106 (7.4%)	758 (52.6%)
**Fragmented**	39 (2.7%)	50 (3.5%)	32 (2.2%)
**Missing**	82 (5.7%)	552 (38.3%)	73 (5.1%)

[a] BUSCO metrics are based on 1,440 plant orthogroups using the final genome assembly as input.

Supplementary Table 5. ALLMAPs anchoring statistics for the BC4 male genome assembly.

	**Contigs**			
	**With markers**	**Anchored**	**Oriented**	**Unplaced**
**Markers (unique)**	3725	3703	2551	22
**Markers per Mb**	9.5	9.6	11.3	0.1
**N50 Contigs**	143	142	76	54
**Contigs**	350	335	112	2371
**Contigs with 1 marker**	78	70	4	8
**Contigs with 2 markers**	56	49	9	7
**Contigs with 3 markers**	28	28	4	0
**Contigs with >=4 markers**	188	188	95	0
**Total sequence (Mb)**	391.1 (50.6%)	385.6 (49.9%)	225.8 (29.2%)	386.7(50.1%)

Supplementary Table 6. Lengths of sequences anchored to linkage groups in the BC4 male date palm assembly.

**Linkage groups[a]**	**Length (bp)**
**1**	40,814,151
**4**	33,281,721
**8**	31,698,078
**11**	29,487,722
**2**	29,301,675
**3**	24,755,689
**14**	24,628,924
**9**	22,757,669
**5**	18,619,412
**6**	18,596,258
**7**	16,639,383
**17**	16,126,437
**10**	15,825,318
**12**	14,769,854
**16**	13,553,361
**13**	12,891,333
**15**	12,030,914
**18**	9,812,533

[a]Linkage group numbers are based on assignments in Matthew *et al.* (2014)[4].

Supplementary Table 7. Summary of RNA-Seq libraries used for genome annotation[a].

Tissue	Pairs	Read length	Mb	% mapped
**Leaf**	6,968,616	94	1,310.1	99.72
	10,483,561	94	1,970.9	
	11,467,980	94	2,156.0	
	8,079,969	94	1,519.0	
	5,967,701	94	1,121.9	
**Fruit**	17,586,364	101	3,552.4	99.50
	14,540,233	101	2,937.1	
	20,152,822	101	4,070.9	
	20,128,165	101	4,065.9	
	23,904,260	101	4,828.7	
	19,323,941	101	3,903.4	
	15,308,983	101	3,092.4	
	19,533,149	101	3,945.7	
**Root**	5,860,094	94	1,101.7	99.08
	7,883,042	94	1,482.0	
	5,500,499	94	1,034.1	
**Flower**	56,765,454	101	11,466.6	98.53
**Pollen**	57,249,724	101	11,564.4	99.10

[a] Libraries from fruit, flower and pollen are new to this study.

Supplementary Table 8. Summary of MAKER2 gene annotations of the date palm BC4 male assembly.

	**All models**	**High confidence**
Loci	36,162	28,595
Isoforms	51,395	43,815
**Assigned to chromosomes**:		
Loci	24,029	20,109
Isoforms	36,240	32,311
**Not assigned to chromosomes**:		
Loci	12,133	8,486
Isoforms	15,155	11,504
**Median size (bp):**		
Transcript (genomic)	2994	4183
Protein	249	309
CDS	143	137
Exon	165	159
Intron	340	328
**AED score**:		
<0.2	39.81%	46.70%
⩽0.5	79.91%	93.73%
⩽0.8	84.10%	98.65%
**eAED score**:		
⩽0.2	38.47%	45.13%
⩽0.5	77.23%	90.59%
⩽0.8	82.30%	96.55%

Supplementary Table 9. Summary of repetitive sequences in the BC4 male genome based on RepeatMasker.

		Mb	Repeat #	repeat fraction	genome fraction
Class I (Retrotransposons)					
LTR	Copia	67.80	61,763	16.80	8.78
	Gypsy	49.04	47,509	12.15	6.35
	Retrovirus	0.58	649	0.14	0.07
	Other LTR	87.10	133,160	21.58	11.28
LINE	L1	12.13	14,623	3.01	1.57
	Other LINE	7.79	26,911	1.93	1.01
SINE		1.51	7,937	0.37	0.20
Other Class I		25.38	20,844	6.29	3.29
Class II (DNAt) subclass 1					
TIR	Tc1–Mariner	0.01	2	0.01	0.01
	hAT	3.23	5,009	0.80	0.42
	Mutator	1.49	3,440	0.37	0.19
	PIF–Harbinger	0.17	547	0.04	0.02
	CACTA	1.36	3,896	0.34	0.18
	Other DNAt	8.59	21,222	2.13	1.11
Class II (DNAt) subclass 2					
Helitron		0.49	2,608	0.12	0.06
Other Class II		1.42	3,891	0.35	0.18
Total TEs		268.06	354,011	66.42	34.71
Ribosomal DNA		0.21	232	0.05	0.03
Structural Repeats		28.19	179,350	6.98	3.65
Unclassified		107.10	206,457	26.54	13.87
**Total Repeats**		**403.56**			**52.25**

Supplementary Table 10. Genes located within approximately 100 kb of the GWAS peak for fruit color.

Gene	Start	End	Protein	Description
Pdac_HC_chr4T0136300	23956950	23969919	Pdac_HC_chr4T0136300.1	autophagy-related protein 18f-like
Pdac_HC_chr4T0136400	23976665	23977303	Pdac_HC_chr4T0136400.1	uncharacterized protein LOC103696160
Pdac_HC_chr4T0136500	23993692	23996620	Pdac_HC_chr4T0136500.1	protein ACCELERATED CELL DEATH 6-like
Pdac_HC_chr4T0136800	24019579	24031342	Pdac_HC_chr4T0136800.1	traB domain-containing protein (LOC103717701)
Pdac_HC_chr4T0136900	24031950	24034117	Pdac_HC_chr4T0136900.1	uncharacterized protein LOC103706216
**Pdac_HC_chr4T0137100**	**24051178**	**24054765**	**Pdac_HC_chr4T0137100.1**	**Virescens**
Pdac_HC_chr4T0137200	24060581	24062021	Pdac_HC_chr4T0137200.1	protein TOC75-3, chloroplastic-like
Pdac_HC_chr4T0137300	24064995	24072434	Pdac_HC_chr4T0137300.1	no hits
Pdac_HC_chr4T0137400	24075355	24076002	Pdac_HC_chr4T0137400.1	protein NDR1-like
Pdac_HC_chr4T0137500	24080274	24080933	Pdac_HC_chr4T0137500.1	NDR1/HIN1-like protein 2
Pdac_HC_chr4T0137600	24099290	24104430	Pdac_HC_chr4T0137600.1	peroxidase 57-like
Pdac_HC_chr4T0137800	24124113	24130643	Pdac_HC_chr4T0137800.1	peroxidase 1-like
Pdac_HC_chr4T0137900	24133892	24136713	Pdac_HC_chr4T0137900.1	peroxidase 56-like
Pdac_HC_chr4T0138000	24141428	24141796	Pdac_HC_chr4T0138000.1	probable carboxylesterase 15
Pdac_HC_chr4T0138200	24177755	24179676	Pdac_HC_chr4T0138200.1	peroxidase 1-like

Supplementary Table 11. Manually annotated gene and feature coordinates for *VIR* on linkage group 4 of the BC4 male genome[a]

Allele	Feature	Start	End
VIR^{IM}	Exon 1 (CDS)[b]	24,051,178	24,051,313
	Intron 1[b]	24,051,314	24,051,384
	Exon 2 (CDS)[b]	24,051,385	24,051,514
	Intron 2[b]	24,051,515	24,052,390
	Exon 3 (CDS)	24,052,391	24,052,628
	Ibn Majid (LTR retrotransposon)[c]	24,052,621	24,064,344
	Termination codon[d]	24,052,629	24,052,631
VIR^{+}	Exon 3 (CDS) 5' of *Ibn Majid*	24,052,391	24,052,620
	Exon 3 (CDS) 3' of *Ibn Majid*		
	Termination codon	24,064,527	24,064,529
vir^{saf}	ATG/ATA SNP	24,051,180	24,051,180

[a]Reference assembly coordinates are provided separately for the reference assembly with LTR retrotransposon insertion allele (*VIR*^{IM}) and alternate (non-reference) alleles *VIR*$^{+}$ and *vir*^{saf} sequences.

[b]Coordinates are shared by *VIR*^{IM} and *VIR*$^{+}$

[c]Target site duplicated sequence (ACCAG) is located at 24,052,616-24,052,620 and 24,064,340-24,064344

[d]Premature stop codon

Supplementary Table 12. Differential expression analysis of *Virescens*

dpp[a]	baseMean[b]	log2FoldChange[c]	lfcSE[d]	statistic[e]	*P* value[f]
**45**	4.046569	0.45	0.52	0.87	0.39
**75**	3.948517	-0.43	0.46	-0.93	0.35
**105**	9.776764	-1.44	0.72	-2.01	0.045
**120**	148.6892	-2.67	0.88	-3.04	0.0024
**135**	259.1735	-1.30	0.57	-2.27	0.023

[a]Day post pollination

[b]Mean of normalized counts of all samples (normalized for sequencing depth). Source data are provided as a Source Data file.

[c]The expression in Khalas relative to Khenezi varieties (i.e., a negative values implies higher expression in Khenezi)

[d]Standard error of the log fold change

[e]Wald statistic

[f]Uncorrected *P* value for the purpose of differential expression for analysis of a single gene

Supplementary Table 13. Pearson's correlation coefficients calculated for each pair of sugar-related traits[a].

	**Sucrose**	**Glucose**	**fructose**	**fructose + glucose**	**sucrose/total sugar**	**total sugar**
**sucrose**	1.00					
**glucose**	-0.96***	1.00				
**fructose**	-0.98***	0.99***	1.00			
**fructose + glucose**	-0.98***	1.00***	1.00***	1.00		
**sucrose/total sugar**	1.00***	-0.97***	-0.98***	-0.98***	1.00	
**total sugar**	-0.42***	0.64***	0.58***	0.61***	-0.43***	1.00

***$P < 0.001$

[a]Pearson's correlation coefficients and associated p-values were calculated in *R* software[3] using the function r_{corr} (n=125). Source data are provided as a Source Data file.

Supplementary Table 14. Differential gene expression analysis of sucrose- and reducing-sugar types of date palm using RNA-Seq[a].

Gene ID	baseMean[b]	Log2FoldChange[c]	lfcSE[d]	Statistic[e]	*P* value[f]
CWINV1 Pdac_HC_chr14G0022900	2839.32	2.69	0.56	8.28	1.25×10^{-16}
CWINV3 Pdac_HC_chr14G0023100	2915.86	4.23	0.48	8.97	3.06×10^{-19}
A/N-INV1 Pdac_HC_chr14G0028200	590.07	0.41	0.24	1.72	0.085

[a]The first two rows show *CWINV1* (Pdac_HC_chr14G0022900) and *CWINV3* (Pdac_HC_chr14G0023100), which are respectively the fifth and second most differentially expressed genes. Also shown, third line, are results for *A/N-INV1* (Pdac_HC_chr14G0028200), which is not among the most differentially expressed genes, but included here for comparative purposes. Log2 fold-change (LFC) greater than 0 reflect higher expression in reducing-sugar types, while values < 0 reflect greater expression in sucrose-types. LFC estimates are "shrunken" estimates obtained using the lfcshrink function in DESeq2. Source data are provided as a Source Data file.

[b]Mean of normalized counts of all samples (normalized for sequencing depth)

[c]The expression in reducing-sugar types relative to sucrose-types

[d]Standard error of the log fold change

[e]Wald statistic

[f]Uncorrected *P* value for the purpose of differential expression for analysis of a single gene

Supplementary Table 15. Enzymatic activity per gram of fresh sample of invertase in four date palm varieties[a].

Cultivar	Fruit type	Replicate 1 enzyme activity (background activity)[b]	Replicate 2 enzyme activity (background activity)[b]
**Ajwa**	reducing sugar-type	13.26 (0.00)	17.71 (0.94)
**Barhee**	reducing sugar-type	12.46 (0.00)	16.34 (0.00)
**Nbet Zayed**	sucrose-type	3.97 (0.00)	5.11 (0.00)
**Wanana**	sucrose-type	4.77 (0.00)	3.80 (0.00)

[a]Two replicate assays were conducted per variety.

[b]Background activity is the assayed activity of the crude extract after boiling. Activities are in units/L.

Supplementary References

1. Lipka, A. E. *et al.* GAPIT: Genome association and prediction integrated tool. *Bioinformatics* **28**, 2397–2399 (2012).

2. VanRaden, P. M. Efficient Methods to Compute Genomic Predictions. *J. Dairy Sci.* **91**, 4414–4423 (2008).

3. R Core Team. *R: A Language and Environment for Statistical Computing*. (R Foundation for Statistical Computing, 2015).

4. Mathew, L. S. *et al.* A first genetic map of date palm (*Phoenix dactylifera*) reveals long-range genome structure conservation in the palms. *BMC Genomics* **15**, 285 (2014).
