# Supplementary material for: Genome-wide association mapping of date palm fruit traits
Source: Nat Commun. 2019 Oct 15;10:4680. doi: 10.1038/s41467-019-12604-9 (PMC6794320; doi:10.1038/s41467-019-12604-9)
Supplement: Supplementary file 3 — Reporting summary [file 41467_2019_12604_MOESM3_ESM.pdf]

nature research

Corresponding author(s): Michael D. Purugganan

Last updated by author(s): Sep 4, 2019

Reporting Summary

Nature Research wishes to improve the reproducibility of the work that we publish. This form provides structure for consistency and transparency in reporting. For further information on Nature Research policies, see Authors & Referees and the Editorial Policy Checklist .

Statistics

For all statistical analyses, confirm that the following items are present in the figure legend, table legend, main text, or Methods section.

n/a	Confirmed	
☐	☒	The exact sample size (*n*) for each experimental group/condition, given as a discrete number and unit of measurement
☐	☒	A statement on whether measurements were taken from distinct samples or whether the same sample was measured repeatedly
☐	☒	The statistical test(s) used AND whether they are one- or two-sided<br>*Only common tests should be described solely by name; describe more complex techniques in the Methods section.*
☐	☒	A description of all covariates tested
☐	☒	A description of any assumptions or corrections, such as tests of normality and adjustment for multiple comparisons
☐	☒	A full description of the statistical parameters including central tendency (e.g. means) or other basic estimates (e.g. regression coefficient) AND variation (e.g. standard deviation) or associated estimates of uncertainty (e.g. confidence intervals)
☐	☒	For null hypothesis testing, the test statistic (e.g. *F*, *t*, *r*) with confidence intervals, effect sizes, degrees of freedom and *P* value noted<br>*Give P values as exact values whenever suitable.*
☒	☐	For Bayesian analysis, information on the choice of priors and Markov chain Monte Carlo settings
☒	☐	For hierarchical and complex designs, identification of the appropriate level for tests and full reporting of outcomes
☐	☒	Estimates of effect sizes (e.g. Cohen's *d*, Pearson's *r*), indicating how they were calculated

Our web collection on statistics for biologists contains articles on many of the points above.

Software and code

Policy information about availability of computer code

Data collection	All software used in the collection of data are publicly available (open source or licensed for academic use). Versions are listed in the Methods section of the manuscript.
Data analysis	All software used in the analysis of data are publicly available (open source or licensed for academic use). Versions are listed in the Methods section of the manuscript. The R statistical computing language was used for statistical analysis unless otherwise indicated. A statement to this effect is included in the manuscript.

For manuscripts utilizing custom algorithms or software that are central to the research but not yet described in published literature, software must be made available to editors/reviewers. We strongly encourage code deposition in a community repository (e.g. GitHub). See the Nature Research guidelines for submitting code & software for further information.

Data

Policy information about availability of data

All manuscripts must include a data availability statement. This statement should provide the following information, where applicable:

- Accession codes, unique identifiers, or web links for publicly available datasets
- A list of figures that have associated raw data
- A description of any restrictions on data availability

The genome and various accessory files have been deposited in the Sequence Read Archive (SRA) under NCBI BioProject PRJNA322046 ([https://www.ncbi.nlm.nih.gov/bioproject/322046]). Short read sequencing data from the GWAS panel have been deposited in the SRA under PRJNA505141 ([https://www.ncbi.nlm.nih.gov/sra/PRJNA505141)]. RNA-seq data for differential gene expression experiments have been deposited in the SRA under PRJNA505138 ([https://www.ncbi.nlm.nih.gov/Traces/study1/?acc=PRJNA505138)]. A genome browser was constructed using Tripal (v2.1) and can be explored visually at [https://datepalmgenomehub.abudhabi.nyu.edu]. SNP data for the GWAS have been deposited at the Dryad Digital Repository ([https://datadryad.org/resource/doi:10.5061/dryad.3mc4265]). Phenotype data are available as Supplementary Data 1.

Field-specific reporting

Please select the one below that is the best fit for your research. If you are not sure, read the appropriate sections before making your selection.

- [x] Life sciences
- [ ] Behavioural & social sciences
- [ ] Ecological, evolutionary & environmental sciences

For a reference copy of the document with all sections, see nature.com/documents/nr-reporting-summary-flat.pdf

Life sciences study design

All studies must disclose on these points even when the disclosure is negative.

Sample size	Sample size was determined based on the number of date palm available in the farms (mapping population). A maximum of n=157 palms were phenotyped. Sample sizes for all traits are clearly stated in the manuscript.
Data exclusions	Date palm samples were excluded from the GWAS if the phenotype could not be determined for the particular trait.
Replication	GWAS was performed with multiple different SNP filter call sets. Statistical associations using the criteria outlined in the manuscript were repeatable across call sets. Traits without statistical associations also show no association in the additional filter call sets.
Randomization	Sampling of palm varieties was based on availability for both GWAS and RNA-seq experiments. Fruit sampling for phenotyping and expression analysis was haphazard from a fruit bunch taking precautions to avoid bruised fruit and to sample the same developmental stage across samples. Experimental procedures including RNA-seq and enzyme activity assays were fully randomized.
Blinding	Blinding was not possible for collection of fruit photographs/image, weight and size measurements as the variety of palm trees was known by the individual collecting the data. We do not consider this to be a source of error as all fruits collected were also phenotyped. For sugar, moisture, and acid composition trait data, the phenotypes were collected blind to the palm varieties. For RNA-seq and enzyme data the varities were blind to the researcher.

Reporting for specific materials, systems and methods

We require information from authors about some types of materials, experimental systems and methods used in many studies. Here, indicate whether each material, system or method listed is relevant to your study. If you are not sure if a list item applies to your research, read the appropriate section before selecting a response.

Materials & experimental systems

n/a	Involved in the study	
x		Antibodies
x		Eukaryotic cell lines
x		Palaeontology
x		Animals and other organisms
x		Human research participants
x		Clinical data

Methods

n/a	Involved in the study	
x		ChIP-seq
x		Flow cytometry
x		MRI-based neuroimaging
