# Supplementary material for: Genome-wide association mapping of date palm fruit traits
Source: Nat Commun. 2019 Oct 15;10:4680. doi: 10.1038/s41467-019-12604-9 (PMC6794320; doi:10.1038/s41467-019-12604-9)
Supplement: Supplementary file 4 — Description of Additional Supplementary Files [file 41467_2019_12604_MOESM4_ESM.docx]

**Description of Additional Supplementary Files**

File name: Supplementary Data 1

Description: Sample information for GWAS mapping population and phenotypes

File name: Supplementary Data 2

Description: Coordinates from the Princpal component Analysis (PCA) inferred by Gapit software on 157 date palms

File name: Supplementary Data 3

Description: Kinship matrix inferred by Gapit software on 157 date palms using Van Raden algorithm

File name: Supplementary Data 4

Description: Significant SNPs associated with sex

File name: Supplementary Data 5

Description: Summary of the genome-wide association study

File name: Supplementary Data 6

Description: Genes located in the 1.1 Mb interval on linkage group 14 associated with variation in fruit sugar composition
