# Supplementary material for: Genome-wide association mapping of date palm fruit traits
Source: Nat Commun. 2019 Oct 15;10:4680. doi: 10.1038/s41467-019-12604-9 (PMC6794320; doi:10.1038/s41467-019-12604-9)
Supplement: Supplementary file 2 — Peer Review [file 41467_2019_12604_MOESM2_ESM.pdf]

Reviewers' comments:

Reviewer #1 (Remarks to the Author):

Comments for the manuscript entitled “Genome-wide association mapping in date palms reveals parallel evolution of fruit traits”

Date palm is an important fruit crop across the Middle East and Northern Africa. The authors improved the reference genome of date palm using long read sequencing technologies. Even if the species is heterozygous, the contig N50 reached to 897 kb. Based on the new assembly, GWAS analysis was then performed on 21 fruit traits. Clear GWAS hits were discovered for sex determination, fruit color, and sugar composition. The manuscript did a great job demonstrating successful GWAS analysis in a non-model species. I have seen many GWAS in non-model species, in which GWAS is often less satisfying due to the limited sample size, phenotyping inconsistency, low-quality reference, genotyping error, etc. Given the sample size is small, I was a little surprised to see those GWAS spikes even for these less complex traits. In general, the manuscript was clearly written and the analytical methods were sound. I have only a few minor concerns about the paper.

1. The title of the manuscript mentioned parallel evolution, but the manuscript did not focus much on the parallel evolution of fruit traits. The only evidence I can find is that the fruit color gene VIR identified in this study has an orthologue in oil palm. In the abstract, the author wrote “We also report a significant association between fruit sugar composition and a genomic region that contains a neutral/alkaline invertase gene. Homologues of these genes have been implicated in fruit color and sugar composition traits in fruits of other crop species, demonstrating the importance of parallel evolution in domestication and the evolution of crop species.” Maybe I have missed something, I did not see evidence of parallel evolution of sugar composition. I would suggest to revise the title to a more suitable one.
2. The new assembly has longer N50 length, and is more complete, but the order of assembled contigs was not examined. Are there any well assembled BACs for a comparison? Or can the author check LD breaks in these contigs?
3. Sex determination should be an extremely simple trait. In Fig. 2c (zoom in), there are two peaks. Is it due to genetics or assembly error?
4. In the fruit color section, I do not understand the color system. “a” represents red/green (I would assume this is a ratio of the two colors) and “b” represents yellow/blue, so “a/b” is red/green/yellow/blue? What does “a/b” mean and why the authors use it as the response variable?

Reviewer #2 (Remarks to the Author):

This paper was an enormous pleasure to read. In my experience, it is incredibly rare to be asked to review such a complete, cohesive, thorough and well-written paper. I commend the authors for taking the time not only to complete and document the analyses thoroughly, but also to tell a great story about the domestication history of this fascinating crop.

The major results of this paper include a novel assembly and annotation of the date palm genome, together with a GWAS of dozens of traits across ~170 samples using whole-genome sequence data. The major findings from the GWAS include the identification of two loci, one involved in sex determination and the other involved in colour. The in-depth analyses of these loci revealed their mode of action, and in the case of sex determination, convincing evidence is provided of the precise

molecular changes that cause the male/female dichotomy in date palm. The findings are novel and of interest to the scientific community. The authors are correct in noting that there is a deficit of these kinds of deep genomic insights in perennial crops relative to annual crops. For this reason, I feel that this paper is particularly important to the field of agricultural genomics.

My expertise lies in the collection and analysis of the next-generation DNA sequence data for the purposes of population genetic analysis and GWAS. I cannot comment on genome assembly, genome annotation, or the RNA-seq analyses as these are outside of my area of expertise.

The writing is excellent. In particular, the Introduction provides an excellent backdrop to the findings of the paper. The figures are clear and concise, and the analyses are sufficiently well described in the methods to enable the reader to fully understand what was done. I have very little to comment on since I found this paper to be exceptionally well done.

Minor comments:
Why did the authors use a 5.5% downsampled SNP set for the GWAS? Was it for computational efficiency or reduction in multiple testing threshold? Whatever the case, it may be worth simply state the reasoning in the Methods section.

Fig 4A – I think "Khalas" and "Soukar Iraqi" may be clearer as X axis labels here instead of "reducingsucrose". For the other axes that use "reducing" and "sucrose", perhaps turn them 45 degrees for clarity.

The word "gender" is used instead of "sex" at one point and I'm unsure if that is appropriate when talking about plants.

In my opinion, the authors may have missed GWAS hits not only because of their relatively small sample size, but because they significantly downsampled the SNP set before running the initial GWASs. The aim should be to ensure, if at all possible, that the SNPs used in the GWAS are in high LD with potentially causal alleles. The authors show an LD decay curve and provide a "half decay distance", but from the decay curve it is difficult for me to evaluate their actual power to detect a real GWAS hit in a high recombination region of the genome where LD decays unusally rapidly, for example. With only 1 SNP per ~2kb in the downsampled GWAS SNP set, you may very well be missing hits that would be detected if the full SNP set was used. It is customary in human GWAS (and other model organisms) to use SNP sets in GWAS that are even larger than the full SNP set here (>7M SNPs). For the purposes of the present study, I feel that the authors have done a great job of identifying major effect loci and uncovering their mode of action, etc… However, it just may be the case that there are other loci in their data set waiting to be discovered and I encourage them to have a closer look by running the GWAS with the full SNP set to see what may come up. By no means am I recommending this to be included in the current manuscript: the authors have demonstrated that their SNP downsampling is appropriate for picking up two major loci. However, if I had whole genome sequence data from my apple GWAS population, I'd definitely test every single markers' association with every phenotype available.

There are missing SRA identifiers (L433 and 434/genome annotation section)

Include the threshold for excess heterozygosity during SNP filtering?

The title is a little odd given that the parallel evolution aspect only gets mentioned in the last few sentences.

Review: Sean Myles

Reviewer #3 (Remarks to the Author):

This study presents an improved genome sequence of date palm assembled from PacBio long reads and GWAS of important fruit quality traits using a panel consisting of a total of 157 date palm accessions that were grown in two farms in United Arab Emirates. The authors identified genome loci highly associated with fruit color and fruit sugar composition, and from these loci, they further identified candidate genes underlying these traits. Of particular interest, mutations in an R2R3-MYB transcription factor were identified to affect fruit color in date palm.

1. "reveals parallel evolution of fruit traits" in the title: I don't think this manuscript talks much about the parallel evolution of fruit traits. The identified R2R3-MYB TF did provide evidence to support the parallel evolution of fruit color in different species, but this information is not sufficient enough to "reveal parallel evolution of fruit traits". In the case of the invertase, homology information was instead used to select this candidate gene, and as noted by the authors (Line 345-353), its role in regulating the sugar composition in date palm remains to be determined. "Genome-wide association mapping in date palms dissects the genetic architecture of fruit color and sugar composition" could be more appropriate, as these are the only two traits with positive GWAS results (besides sex determination) that were presented in the manuscript.

2. Line 164-169: Pseudomolecules were constructed using a low-resolution genetic map. However, only about half of the genome assembly was able to be included in the pseudomolecules. This would limit the usage of this reference genome. With the recent technology advances (e.g., Hi-C), a much more complete chromosome-level assembly can be feasibly obtained, which would substantially benefit the research and breeding of date palm. In addition, according to Fig. S2a, there are quite a few inconsistencies between the genetic map and the assembly. Careful checking is needed to make sure the inconsistencies are not due to assembly errors.

3. Line 237-238: Fig. 3b shows "Fruit color measured by a color index (a/b) as a function of anthocyanin level" (copied from the figure legend). It is interesting that a significant association signal was identified for fruit color using the color index (a/b), while no associations were identified for the anthocyanin level (Fig. S7). Please discuss or comment on this.

4. In a number of places data presentation can be improved and figure/table citations are not clear. Here are several examples:
4.1 Table 1 does not provide much more information than what already described in the manuscript or in Table S1. A table listing the statistics of this genome assembly and the other two previously published genomes would provide much more useful information.
4.2 Figure 1 seems boring to me. The information presented in this figure was either clearly described in the manuscript or not important (especially Fig. 1b-f). Either remove this figure or put it in supplement, or expand Fig. 1g to include more tracks such as repeat density, gene expression profile, SNP density…
4.3 Fig. 4c and Table 2 contain similar information. I would suggest to remove Fig. 4c. In addition, I don't think this information is very important so Table 2 can be put in supplement.
4.4 Table S9: genome resequencing information for the date palm accessions should be added to this table.
4.5 Table S13: Please also add the numbers of significant SNPs identified using the full SNP dataset.
4.6 Line 142-187: In this entire section, "Fig. 1" was cited instead of the exact subfigures of Fig. 1, which has a-g.
4.7 Line 167-169: not sure why Fig. S2 was cited here as it is unrelated to this statement.
4.8 Line 197: Fig. S4 was cited here but no further description was provided for this figure, which shows the clustering of the accessions.
4.9 Line 239-240: not sure why Fig. S7 was cited here as this figure does not show the results of GWAS on the ratio of a/b.

5. Line 157: couldn't find the document of "Supplementary Online Material".

6. Line 429, "Only one contig aligned to two different LGs and had to be split": this sentence is hard to understand.

7. Line 459-460: Fig. S1 does not provide much useful information and together with this sentence should be removed.

8. Line 461: v2 is outdated. Please consider using BUSCO v3 with the most recent plant dataset (e.g., "Embryophyta odb 10").

9. Line 482-483: GIS information of the two farms could be provided.

10. Line 489: "volatile profiling" was mentioned here but I couldn't find any analysis related to this.

11. Line 521: "an excess in heterozygosity" is too vague. Need to clearly define this.

12. Fig. S3: Labels of both x-axis and y-axis are incomplete and not clear, e.g., "Ch…", "c…". What is "Pdac_Barhee_chr_unan_cp_180126"?

Reviewer #4 (Remarks to the Author):

Genome wide association mapping in date palms reveals parallel evolution of fruits

There are several aspects to this study. Firstly, Hazzouri and colleagues produce an excellent high quality genome assembly for Phoenix by using the PacBio long read system, making a huge improvement on the current genome assembly. Secondly, the use this assembly to develop a genome wide association (GWAS) approach as a proof of principle for long generation organisms that cannot be effectively studied using classic QTL mapping approaches. The team then test the GWAS on a set of 157 short read genomes supplied by a single orchard. To do this they select a known locus/trait association, sex determination and successfully recover the sex determination region as evidenced by low associated recombination as expected in a sex determination region. Thirdly, the team then go on to use the GWAS approach to recover genes associated with fruit traits. They recover a clear signal on fruit colour, recovering the same gene using several different trait measures of colour, VIRISCENS. The group had previously recovered this gene in their previous 2015 Nat Comms study, where the observation is made that this homolog is also causative of colour control in other fruit crops, hence the parallel evolution referenced in the title and also described in the 2015 study. The group identify the copia element, as with the 2015 study, as responsible for causing a disruption of the gene with dominant/partial dominant effect, although in this study they recover more sequence information. New to this study is the discovery of a second, recessive, causative SNP at the same locus. Entirely new to this study is the recovery of GWAS peaks associated with sugar metabolism determining taste type on the basis of reducing sugar/sucrose ratio. This signal is less clean than the previous signals spanning a relatively large region which contains a likely candidate, inv3, which they demonstrate is less expressed in sucrose types so explanatory for the reduced conversion of sucrose to glucose and fructose.

There is much to like about this work, although in terms of learning more about date palm evolution it is incremental to their previous work. The study clearly demonstrates the value of GWAS in this context, which I think is where the strength of the paper lies. The actual findings about date palm are

more incremental and seem to largely echo previous findings. The sugar metabolism evidence is still a bit short of convincing.

Specific comments:
In regard to the sugar metabolism, no sequence data evidence is presented to explore possible underlying causative differences, which is surprising.

The inv3 activity does not seem to scale to reducing sugar content. Perhaps there is another gene involved – not necessarily one underlying gene here, which the messy GWAS signal would seem to support (Fig 4f/g). Some more analysis/discussion around this point would be appropriate.

It is a pity that there is no RNA time course data presented for inv3 expression of a non-reducing sugar variety, it is assumed that expression is universally restricted to the khalal stage (Fig 4e)

It is also a pity that there isn't RNA data presented for the saf mutant expression – as I understand it we only see the copia element adjusted expression levels (Fig 3f)

RESPONSE TO REVIEWERS

We thank the four very thoughtful and constructive reviewers. This was certainly a case when the reviews helped substantially improve the paper! Here is how we addressed the comments.

Reviewer #1:

1. The title of the manuscript mentioned parallel evolution, but the manuscript did not focus much on the parallel evolution of fruit traits. The only evidence I can find is that the fruit color gene VIR identified in this study has an orthologue in oil palm. In the abstract, the author wrote "We also report a significant association between fruit sugar composition and a genomic region that contains a neutral/alkaline invertase gene. Homologues of these genes have been implicated in fruit color and sugar composition traits in fruits of other crop species, demonstrating the importance of parallel evolution in domestication and the evolution of crop species." Maybe I have missed something, I did not see evidence of parallel evolution of sugar composition. I would suggest to revise the title to a more suitable one.

We agree and have changed the title simply to "Genome-wide association mapping of date palm fruit traits."

2. The new assembly has longer N50 length, and is more complete, but the order of assembled contigs was not examined. Are there any well assembled BACs for a comparison? Or can the author check LD breaks in these contigs?

Unfortunately, there are no BAC sequences that are publicly available where we can check. In general, PacBio assemblies are accepted as good assemblies. The one issue that could arise are chimeric contigs, of which we have only identified one. This does raise a point – we have an assembly that is considered good, but maybe since we have not checked it against other data we should refrain from calling it a "reference" genome. We have removed calling it a "reference" and simply a "long-read genome assembly"

3. Sex determination should be an extremely simple trait. In Fig. 2c (zoom in), there are two peaks. Is it due to genetics or assembly error?

Yes, we have noticed this but we remain unsure as to whether these are indeed two peaks, a misassembly or some statistical fluke. The LD in the region is high, which does suggest that these act as one locus. We have added a phrase that point out these possibilities.

4. In the fruit color section, I do not understand the color system. "a" represents red/green (I would assume this is a ratio of the two colors) and "b" represents yellow/blue, so "a/b" is red/green/yellow/blue? What does "a/b" mean and why the authors use it as the response variable?

a and b are chromatic descriptors with a expressing green to red and b expressing blue to yellow. Because we want to account for a gradient in color from yellow to red, the ratio is more

appropriate, as explained in the Results, lines 240-241 and in the Methods section, lines 628-630. Note that although we focused on the ratio a/b in the Results, we also ran the association analysis on other color parameters, including a and b alone and they yielded the same results, namely a single significant region on LG4, as shown in Supplementary Figure 8.

Reviewer #2:

1. Why did the authors use a 5.5% downsampled SNP set for the GWAS? Was it for computational efficiency or reduction in multiple testing threshold? Whatever the case, it may be worth simply state the reasoning in the Methods section.

As recommended, we have added a sentence in the Methods that indicate that we wanted to both minimize multiple testing issues while preserving dense SNP coverage, and improve computational efficiency (lines 672-674). Note that as explained lines 680-682, we further performed a second GWAS analysis using the full SNP set on linkage groups where significant SNPs were identified for a particular trait.

2. Fig 4A – I think “Khalas” and “Soukar Iraqi” may be clearer as X axis labels here instead of “reducingsucrose”. For the other axes that use "reducing" and "sucrose", perhaps turn them 45 degrees for clarity.

Given the new results on invertase, we have added a new figure and ensured that the reviewers suggestions were carried out.

3. The word “gender” is used instead of “sex” at one point and I’m unsure if that is appropriate when talking about plants.

We agree and have change the word to “sex”.

4. In my opinion, the authors may have missed GWAS hits not only because of their relatively small sample size, but because they significantly downsampled the SNP set before running the initial GWASs. The aim should be to ensure, if at all possible, that the SNPs used in the GWAS are in high LD with potentially causal alleles. The authors show an LD decay curve and provide a “half decay distance”, but from the decay curve it is difficult for me to evaluate their actual power to detect a real GWAS hit in a high recombination region of the genome where LD decays unusally rapidly, for example. With only 1 SNP per ~2kb in the downsampled GWAS SNP set, you may very well be missing hits that would be detected if the full SNP set was used. It is customary in human GWAS (and other model organisms) to use SNP sets in GWAS that are even larger than the full SNP set here (>7M SNPs). For the purposes of the present study, I feel that the authors have done a great job of identifying major effect loci and uncovering their mode of action, etc… However, it just may be the case that there are other loci in their data set waiting to be discovered and I encourage them to have a closer look by running the GWAS with the full SNP set to see what may come up. By no means am I recommending this to be included in the current manuscript: the authors have demonstrated that their SNP downsampling is appropriate for picking up two major loci. However, if I had whole

genome sequence data from my apple GWAS population, I'd definitely test every single markers' association with every phenotype available.

We thank the reviewer, and we will re-run everything as an exploratory analysis. The reviewer is right in that we should not include this in the paper, as it will likely have too many redundant SNPs. We chose the downsampled SNP set carefully so that the SNP coverage would be dense – given the decay distance of ~20 kb, we should have ~10 SNPs per mean LD block.

5. There are missing SRA identifiers (L433 and 434/genome annotation section)

We removed these mentions and provided SRA numbers in the Data availability section.

6. Include the threshold for excess heterozygosity during SNP filtering?

We have added this information in the Methods.

7. The title is a little odd given that the parallel evolution aspect only gets mentioned in the last few sentences.

We agree and have changed the title.

Reviewer #3:

1. “reveals parallel evolution of fruit traits” in the title: I don’t think this manuscript talks much about the parallel evolution of fruit traits. The identified R2R3-MYB TF did provide evidence to support the parallel evolution of fruit color in different species, but this information is not sufficient enough to “reveal parallel evolution of fruit traits”. In the case of the invertase, homology information was instead used to select this candidate gene, and as noted by the authors (Line 345-353), its role in regulating the sugar composition in date palm remains to be determined. “Genome-wide association mapping in date palms dissects the genetic architecture of fruit color and sugar composition” could be more appropriate, as these are the only two traits with positive GWAS results (besides sex determination) that were presented in the manuscript.

We agree and have changed the title to “Genome-wide association mapping of date palm fruit traits.” Although we only obtained significant results for fruit color and sugar, we did do GWAS mapping on 21 fruit traits. The negative results for the other traits are nevertheless instructive, and we think this more expansive title is appropriate.

2. Line 164-169: Pseudomolecules were constructed using a low-resolution genetic map. However, only about half of the genome assembly was able to be included in the pseudomolecules. This would limit the usage of this reference genome. With the recent technology advances (e.g., Hi-C), a much more complete chromosome-level assembly can be feasibly obtained, which would substantially benefit the research and breeding of date palm. In addition, according to Fig. S2a, there are quite a few inconsistencies between the genetic map

and the assembly. Careful checking is needed to make sure the inconsistencies are not due to assembly errors.

We agree that it would be good to ensure consistency with the genetic mapping, as well as other checks for assembly quality. We indeed are planning to get both Hi-C data and a denser genetic map, but these will take a year to finalize. Because of this, as discussed above, we have stopped calling this a "reference" genome and instead call this simply a "long-read genome assembly."

3. Line 237-238: Fig. 3b shows "Fruit color measured by a color index (a/b) as a function of anthocyanin level" (copied from the figure legend). It is interesting that a significant association signal was identified for fruit color using the color index (a/b), while no associations were identified for the anthocyanin level (Fig. S7). Please discuss or comment on this.

We have added a sentence that says: "While anthocyanin content in these genotypic classes are consistent with functional variation at VIR, GWAS on anthocyanin content does not lead to significant hits, possibly due to the greater variability of anthocyanin level measurements that result in reduced power to detect GWAS peaks." (lines 275-279).

4. In a number of places data presentation can be improved and figure/table citations are not clear. Here are several examples:
4.1 Table 1 does not provide much more information than what already described in the manuscript or in Table S1. A table listing the statistics of this genome assembly and the other two previously published genomes would provide much more useful information.

Table 1 was a nice summary of the assembly statistics and rather than deleting it we moved it to the supplement (now Supplementary Table 1). As suggested by the reviewer, the Table 1 now shows statistics of this new assembly along with statistics from the two previously published genomes.

4.2 Figure 1 seems boring to me. The information presented in this figure was either clearly described in the manuscript or not important (especially Fig. 1b-f). Either remove this figure or put it in supplement, or expand Fig. 1g to include more tracks such as repeat density, gene expression profile, SNP density…

We understand the reviewer's concern. However, we disagree that this figure is not important. This figure describes how the genome was assembled and key information about the assembly. Given other comments that we should move Table 1 to the supplement (which we did), we believe that leaving Figure 1 in the main text is critical.

4.3 Fig. 4c and Table 2 contain similar information. I would suggest to remove Fig. 4c. In addition, I don't think this information is very important so Table 2 can be put in supplement.

This table provides additional information that is not given in Fig 4c, namely statistical significance and additional correlation. To address the reviewer's comment, we moved the Table to the supplement (it is now Supplementary Table 18), as it provides additional correlations.

4.4 Table S9: genome resequencing information for the date palm accessions should be added to this table.

In the Supplementary Table 9 (now Supplementary Table 10), we added the number of reads and the average depth for each accession.

4.5 Table S13: Please also add the numbers of significant SNPs identified using the full SNP dataset.

The numbers of significant SNPs identified using the full SNP set are already in the Supplementary Table 13 (now Supplementary Table 14). We ran the GWAS analysis using the full SNP set on particular linkage groups where significant SNPs were identified during the preliminary analysis using the downsampled dataset.

4.6 Line 142-187: In this entire section, “Fig. 1” was cited instead of the exact subfigures of Fig. 1, which has a-g.

Done

4.7 Line 167-169: not sure why Fig. S2 was cited here as it is unrelated to this statement.

Indeed, the Supplementary Figure 2 was related to the previous sentence so we moved its mention to the previous sentence.

4.8 Line 197: Fig. S4 was cited here but no further description was provided for this figure, which shows the clustering of the accessions.

This figure shows that there is no obvious structure related to the origins of the accession (two farms). It also shows that these are diverse accessions, that are not inbred. To answer the reviewer’s concern, we explained this in the Results, lines 196-198.

4.9 Line 239-240: not sure why Fig. S7 was cited here as this figure does not show the results of GWAS on the ratio of a/b.

The reviewer is right, there was an error and we thus removed the mention of the figure.

5. Line 157: couldn’t find the document of “Supplementary Online Material”.

It was a mistake and we removed this.

6. Line 429, “Only one contig aligned to two different LGs and had to be split”: this sentence is hard to understand.

We have rewritten the sentence (lines 500-503).

7. Line 459-460: Fig. S1 does not provide much useful information and together with this sentence should be removed.

We removed this figure.

8. Line 461: v2 is outdated. Please consider using BUSCO v3 with the most recent plant dataset (e.g., “Embryophyta odb 10”).

We have done so and modified the text accordingly: in the Results, Methods, Fig. 1f, Supplementary Table 1 and Supplementary Table 4.

9. Line 482-483: GIS information of the two farms could be provided.

Done (lines 557-560).

10. Line 489: “volatile profiling” was mentioned here but I couldn’t find any analysis related to this.

Changed to “organic acid”.

11. Line 521: “an excess in heterozygosity” is too vague. Need to clearly define this.

We indeed could have been more specific and thus modified the Methods section to explain how we filtered based on excess in heterozygosity.

12. Fig. S3: Labels of both x-axis and y-axis are incomplete and not clear, e.g., “Ch…”, “c…”. What is “Pdac_Barhee_chr_unan_cp_180126”?

We modified the Figure to make it clearer.

Reviewer #4:

1. In regard to the sugar metabolism, no sequence data evidence is presented to explore possible underlying causative differences, which is surprising.

During the revision process, we have indeed identified two very promising deletions – a ~5 kb deletion proximal to our invertase gene, plus two other invertase genes at the edge of the GWAS peak which contains a 40-kb deletion that renders one invertase non-functional and may also impact downstream regulatory regions of another. As such, the section on the sugar traits has been rewritten to include these added findings, as well as a new figure.

2. The inv3 activity does not seem to scale to reducing sugar content. Perhaps there is another gene involved – not necessarily one underlying gene here, which the messy GWAS signal would seem to support (Fig 4f/g). Some more analysis/discussion around this point would be appropriate.

See #1 above. The original inv3 is now called A/N-INV1 in our revision. We should note that in the revision, we report a P < 0.08 in expression differences for the gene rather than the P < 0.029 in the previous version. This because we changed the workflow to map genes given the multiple genes we are now testing, plus the previous version was a one-sided test (H1: gene expression is lower in sucrose varieties) vs. the now two-sided test (H1: gene expression is different between sucrose and reducing sugar varieties).

3. It is a pity that there is no RNA time course data presented for inv3 expression of a non-reducing sugar variety, it is assumed that expression is universally restricted to the khalal stage (Fig 4e)

We agree that this would have been nice to present, but logistics prevented us from getting a time series of the non-reducing varieties. However, we have now added time course data for two cell wall invertase genes that we now think are good candidate causal genes to sugar variation.

4. It is also a pity that there isn't RNA data presented for the saf mutant expression – as I understand it we only see the copia element adjusted expression levels (Fig 3f)

We did not have a fruit developmental sample with the appropriate genotype that could be used in this time series profile. Nevertheless, for the vir^{saf} *allele, this is not really pertinent: if the gene is not expressed, then it is a loss-of-function allele, but if it is expressed, the translation initiation mutation renders it a loss-of-function allele as well. For the copia insertion allele, however, it was necessary to show expression to explain the dominant/semi-dominant negative effect.*

REVIEWERS' COMMENTS:

Reviewer #1 (Remarks to the Author):

Comments for the manuscript entitled “Genome-wide association mapping of date palm fruit traits”

A high-quality genome assembly is critical for trait dissection in non-model species. The manuscript by Hazzouri et al. provided a useful resource by improving date palm genome with long reads and illustrated GWAS analysis on key traits. The authors took my concerns into account and revised manuscript properly. All the questions were well addressed. I do not more questions about their work.

Reviewer #2 (Remarks to the Author):

The authors have addressed all of the concerns and I feel that the manuscript is now suitable for publication.

Reviewer: Sean Myles

Reviewer #3 (Remarks to the Author):

I found that the authors did not perform any additional analyses or experiments to improve the assembled date palm genome. Instead, they just changed calling the assembled genome from “reference genome” to “long-read genome assembly”. I think this will substantially decrease the significance of this manuscript. Nowadays, high-quality chromosome level genome assemblies of plant species should be required for publications in high-profile journals such as this one. Only 49.9% anchored and 29.2% oriented are far below the standard of genome assemblies recently published in high-profile journals. Furthermore, it is worth noting that current algorithms for PacBio long read assembly still have much room to improve, especially for heterozygous plant species like date palm. Therefore, additional data that can be used to correct and support the assembly are needed. A large number of inconsistencies between the genetic map and the assembly as shown in Fig. S1 raises concern on potential mis-assemblies in this genome.

In my opinion, the GWAS analyses performed in this study did not provide much novel information as most of the GWAS failed to yield positive results probably due to the small population size. The candidate causative gene and variation identified for fruit color seem just confirming their previous findings. For fruit sugar composition, the two deletions identified as causative variations are not very convincing as I suspect other variations (SNPs, small indels, SVs) with similar patterns could be identified in this region. In addition, I am still intrigued that no GWAS signal was detected for anthocyanin levels. This may reflect the low detection power of the GWAS here with a small population size.

Due to the shift of the focus of the manuscript from “parallel evolution” to “GWAS”, I believe in the revised manuscript the introduction section was not well developed. For example, in the first paragraph the authors raised the question about “the evolutionary genetic basis of the origin and diversification of perennial crops such as fruit tree species” but this question was not addressed in this study at all…

Genome size estimation: 774 Mb based on k-mer (Line 489) and 870-899 Mb (Line 154) based on flow cytometry. Quite large difference. Which one we should believe?

Line 556, “Mapping population”: “GWAS panel” could be more appropriate.

Line 599 and 606: “VCFtools”, “vcftools”, please be consistent. Reference citation should be added to Line 599.

Table 1: Change “N50 (Mb)” to “N50 (Kb)”.

Reviewer #4 (Remarks to the Author):

Hazzouri and colleagues have responded comprehensively to the reviewers' comments, and adjusted their manuscript accordingly. I am satisfied that this, with the new title better reflects their work and is now acceptable.

REVIEWERS' COMMENTS:

Reviewer #1 (Remarks to the Author):

Comments for the manuscript entitled “Genome-wide association mapping of date palm fruit traits”

A high-quality genome assembly is critical for trait dissection in non-model species. The manuscript by Hazzouri et al. provided a useful resource by improving date palm genome with long reads and illustrated GWAS analysis on key traits. The authors took my concerns into account and revised manuscript properly. All the questions were well addressed. I do not more questions about their work.

We thank the reviewer for their constructive comments.

Reviewer #2 (Remarks to the Author):

The authors have addressed all of the concerns and I feel that the manuscript is now suitable for publication.

Reviewer: Sean Myles

We thank the reviewer for his constructive comments.

Reviewer #3 (Remarks to the Author):

I found that the authors did not perform any additional analyses or experiments to improve the assembled date palm genome. Instead, they just changed calling the assembled genome from “reference genome” to “long-read genome assembly”. I think this will substantially decrease the significance of this manuscript. Nowadays, high-quality chromosome level genome assemblies of plant species should be required for publications in high-profile journals such as this one. Only 49.9% anchored and 29.2% oriented are far below the standard of genome assemblies recently published in high-profile journals. Furthermore, it is worth noting that current algorithms for PacBio long read assembly still have much room to improve, especially for heterozygous plant species like date palm. Therefore, additional data that can be used to correct and support the assembly are needed. A large number of inconsistencies between the genetic map and the assembly as shown in Fig. S1 raises concern on potential mis-assemblies in this genome.

We thank the reviewer for his comments.

We did redo the BUSCO analyses, and in our previous response we pointed out our work to check on assembly integrity. We feel that for our needs – use of a genome assembly for GWAS mapping – our assembly is of sufficient quality. As we have shown, this assembly is substantially improved compared to two current reference genome assemblies for this crop species, and while

not perfect, we have also shown that this new assembly now allows GWAS mapping in this species. We feel that these two points in themselves show the importance of our results.

In my opinion, the GWAS analyses performed in this study did not provide much novel information as most of the GWAS failed to yield positive results probably due to the small population size. The candidate causative gene and variation identified for fruit color seem just confirming their previous findings. For fruit sugar composition, the two deletions identified as causative variations are not very convincing as I suspect other variations (SNPs, small indels, SVs) with similar patterns could be identified in this region. In addition, I am still intrigued that no GWAS signal was detected for anthocyanin levels. This may reflect the low detection power of the GWAS here with a small population size.

It should be pointed out that the previous identification of VIR in our previously published work was based on a candidate gene approach and was very tentative. Our GWAS mapping solidifies this as a genomewide confirmation of the importance of this locus in determining fruit color. Moreover, our new data provides information on new alleles (e.g., the start codon mutation), as well as a clear genetic model for fruit color polymorphism. Finally, we feel the invertase structural polymorphisms and its correlation with gene expression and enzyme activity levels provide new information that is of widespread interest to understanding the genetics of sugar composition in fruits.

Due to the shift of the focus of the manuscript from “parallel evolution” to “GWAS”, I believe in the revised manuscript the introduction section was not well developed. For example, in the first paragraph the authors raised the question about “the evolutionary genetic basis of the origin and diversification of perennial crops such as fruit tree species” but this question was not addressed in this study at all…

We feel at this point that the Introduction is entirely appropriate – we make the case for the need for better genome resources in date palm, and how an improved assembly can aid in GWAS mapping. Moreover, our findings on the GWAS mapping does provide a clear insight into crop evolution – specifically crop diversification – which we discuss in our Discussion.

Genome size estimation: 774 Mb based on k-mer (Line 489) and 870-899 Mb (Line 154) based on flow cytometry. Quite large difference. Which one we should believe?

We feel that the flow cytometry results are more robust; it is a direct experimental estimate of genome size, while the k-mer based results are less direct. When we calculate % of genome covered, we use the cytometry results.

Line 556, “Mapping population”: “GWAS panel” could be more appropriate.

We have changed to “GWAS panel” in the paper as appropriate.

Line 599 and 606: “VCFtools”, “vcftools”, please be consistent. Reference citation should be added to Line 599.

Done.

Table 1: Change “N50 (Mb)” to “N50 (Kb)”.

Done.

Reviewer #4 (Remarks to the Author):

Hazzouri and colleagues have responded comprehensively to the reviewers' comments, and adjusted their manuscript accordingly. I am satisfied that this, with the new title better reflects their work and is now acceptable.

We thank the reviewer for their constructive comments.
